# Environmental Conditioning of Skeletal Anomalies Typology and Frequency in Gilthead Seabream (Sparus aurata L., 1758) Juveniles

**DOI:** 10.1371/journal.pone.0055736

**Published:** 2013-02-07

**Authors:** Loredana Prestinicola, Clara Boglione, Pavlos Makridis, Attilio Spanò, Valentina Rimatori, Elisa Palamara, Michele Scardi, Stefano Cataudella

**Affiliations:** 1 Department of Biology, University of Rome “Tor Vergata”, Rome, Italy; 2 Department of Biology, University of Patras, Patras, Greece; The Hebrew University, Israel

## Abstract

In this paper, 981 reared juveniles of gilthead seabream (*Sparus aurata*) were analysed, 721 of which were from a commercial hatchery located in Northern Italy (Venice, Italy) and 260 from the Hellenic Center for Marine Research (Crete, Greece). These individuals were from 4 different egg batches, for a total of 10 different lots. Each egg batch was split into two lots after hatching, and reared with two different methodologies: intensive and semi-intensive. All fish were subjected to processing for skeletal anomaly and meristic count analysis. The aims involved: (1) quantitatively and qualitatively analyzing whether differences in skeletal elements arise between siblings and, if so, what they are; (2) investigating if any skeletal bone tissue/ossification is specifically affected by changing environmental rearing conditions; and (3) contributing to the identification of the best practices for gilthead seabream larval rearing in order to lower the deformity rates, without selections. The results obtained in this study highlighted that: i) in all the semi-intensive lots, the bones having intramembranous ossification showed a consistently lower incidence of anomalies; ii) the same clear pattern was not observed in the skeletal elements whose ossification process requires a cartilaginous precursor. It is thus possible to ameliorate the morphological quality (by reducing the incidence of severe skeletal anomalies and the variability in meristic counts of dermal bones) of reared seabream juveniles by lowering the stocking densities (maximum 16 larvae/L) and increasing the volume of the hatchery rearing tanks (minimum 40 m^3^). Feeding larvae with a wide variety of live (wild) preys seems further to improve juvenile skeletal quality. Additionally, analysis of the morphological quality of juveniles reared under two different semi-intensive conditions, Mesocosm and Large Volumes, highlighted a somewhat greater capacity of Large Volumes to significantly augment the gap with siblings reared in intensive (conventional) modality.

## Introduction

Gilthead seabream (*Sparus aurata* L.) is a species of high commercial value, especially in the Mediterranean region, where it was one of the first species to be intensively cultivated. In the last few years, the drop in the gilthead seabream market price due to overproduction is forcing the aquaculture industry to reduce production costs and enhance fish quality. The latter goal is seriously affected by the presence of skeletal anomalies, one of the most important bottlenecks in current aquaculture production, as they require manual sorting [1]–[2] and are associated with a general lowering of performance (i.e. swimming ability, conversion index, growth rate, survival, and susceptibility to stress, pathogens and bacteria) [1], [3]–[14]. A variable percentage of 15 to 50% of gilthead seabream juveniles with severe anomalies is actually totally culled out of the productive cycle at the end of the hatchery phase, depending on the rearing methodology followed [2]. However, no farm today can claim a routine production of 100% non-deformed fish, also because the early assessment of severe anomalies is often difficult as they begin as slight aberrations of the internal elements that only later can develop into more severe abnormalities affecting the external body shape. The presence of severely deformed reared fish can cause consumers to lose confidence in aquaculture products [15] and reduce the commercial value of the reared lots [10]–[11], [13]–[14], [16]–[24]. Even automatic fillet processing is impaired by the presence of vertebral deformities, thus reducing economic return [25].

The presence of skeletal anomalies in reared fish is generically attributable to a general lowering of individual homeostasis (the tendency of a biological system to resist change and to maintain itself in a state of stable equilibrium, according to Allaby [26]), i.e. the capacity to buffer variations in the external (environmental) and internal (genetic stress) conditions (through canalisation and developmental stability), thus allowing the expression of deviated ontogenetic and growth processes, such as anatomical anomalies, fluctuating asymmetry, altered meristic counts and anomalous pigmentation [27]. Accordingly, all these anomalies may be considered as developmental disturbances, indicative of the presence of inappropriate rearing conditions [11], [14], [20], [28]–[34] or genetic impairment. According to the available literature, each environmental (biotic and abiotic) factor/parameter (oxygen, temperature, pH, stocking density, water flow, CO_2_, rearing volumes, inappropriate alimentation, heavy metals, bacteria, parasites, toxicants …), if at a non-optimal level for the reared species or life stage, can cause skeletal anomalies in reared fishes [13]–[14], [16]–[17], [35]–[38]. According to Hough [39], the minimum estimate of the annual loss due to deformed fish is more than € 50,000,000/year for European aquaculture, and a reduction of 50% in deformed fish rate could save € 25,000,000/year, increase production and profitability and enhance aquaculture reputation.

In this scenario, a more profound knowledge of how skeletal anomaly onset, typology or incidence can be modulated by environmental conditions could be of great and practical help in improving the quality of farmed juveniles. It should however be considered that: i) various non genetic factors can induce the same skeletal anomaly in different species; ii) the same causative factor can induce different skeletal anomalies in diverse fish species [2]; iii) skeletal anomalies may be induced by different factors in different ‘cohorts’ of the same species [40]; iv) the same causative factor may provoke a higher incidence of anomalies in some skeletal elements, but not in others, having the same bone type and ossification, in the same individual [41]; v) some skeletal anomalies arise only under extreme rearing conditions [42]. Moreover, our understanding of the cause of skeletal anomalies in reared fish is also hampered by the fact that Teleosts present an exceptional diversity of skeletal tissues compared to other Vertebrates (tetrapods), and also between advanced and basal Teleosts: the differences refer to original germ layer, bone and cartilage tissues, type of ossification and evolutionary history [13],[43]. Consequently, inferences from studies carried out on other Vertebrates can be used but only after thorough confirmation in the different reared finfish species.

Traditionally, three different rearing approaches are followed in the framework of the Mediterranean aquaculture of gilthead seabream: intensive, semi-intensive, and extensive. The first accounts for most of gilthead seabream production; the latter is typical of coastal lagoon management (for instance, Italian *valli*) and is rarely applied to larvae and juveniles. Semi-intensive rearing is still occasionally applied in commercial farms, even though it has been demonstrated to be capable of producing juveniles of higher morphological quality [17], [44]–[47] as it requires larger spaces for the larval rearing tanks.

This study analyzed different lots of gilthead seabream juveniles originating from the same egg batch, but reared under different conditions for the purpose of: (1) quantitatively and qualitatively analyzing whether differences in skeletal elements (shape and number) occur and, if so, which are the most common; (2) investigating if a relationship exists between ossification typology and environmentally-induced skeletal anomalies; and (3) identifying the best practices for gilthead seabream larval rearing in order to obtain lower deformity rates.

## Materials and Methods

A total of 981 reared juveniles of gilthead seabream were analysed, 721 of which were from a commercial hatchery located in Northern Italy (Valle Figheri, Venice, Italy), and a further 260 were obtained from the Hellenic Center for Marine Research (Heraklion, Crete, Greece) (Table 1). These individuals were from 4 different egg batches (Groups 1–4), for a total of 10 different lots. Each egg batch/group was split after hatching into two lots, and reared following two different methodologies - intensive and semi-intensive rearing. Only some lots (Group 3: INIT19, INIT18, LVIT04, LVIT05) were sampled at two different ages (Table 1).

**Table 1:** Characteristics of the reared gilthead seabream lots.

Group code	Lot code	Origin	DPH	Rearing condition	n	Mean SL	Range
1	**INIT06**	North-East Italy	77	Intensive	55	18.4	13.5–24
	**LVIT01**		71	Large Volumes^a^	66	24.8	14.5–36
2	**INIT07**	North-East Italy	81	Intensive	123	12.7	9–16.5
	**LVIT02**		79	Large Volumes	122	15.5	13–19
3	**INIT19** *	North-East Italy	63	Intensive	105	13.5	11–17
	**INIT18** *		85		105	30.6	23–41
	**LVIT04** **		64	Large Volumes	40	20.9	17–27
	**LVIT05** **		85		105	32.8	21–41
4	**INGR01**	Crete, Greece	105	Intensive	134	40.6	35–55
	**MEGR04**		105	Mesocosms^b^	126	41.8	36–55
	***Total***				***981***		***9–55***

Each group identifies juveniles from the same egg batch. DPH = days post-hatching; *n* = number of individuals; *SL* = standard length, in mm; *Range* = observed minimum and maximum standard length, for each lot, in mm;

* ,** = same lots but sampled at different ages;

a = *sensu* [48];

b = *sensu* [46].

See the text for further details.

### 1. Ethic Statement

The authorization of the Ethics Committee of the University of Rome Tor Vergata or some other ethical oversight was not required, because sampling was carried out on commercial catches for human consumption.

Following the 2931/29-05-2008 application of Dr. Pascal Divanach, Director of the Institute of Aquaculture and the 2932/30-05-2008 justified report of the Veterinarians A. Grigoriou and Z. Somaras of our Directorate, the facility of Aqualabs A of the Hellenic Centre for Marine Research at Gournes, Heraklion, was registered in the special Directorate book as number: EL 91-BIO-03. Facility address: Aqualabs A- HCMR, Former American Base of Gournes, PO Boc 2214, Postal code 71003, Heraklion, Crete. Administrative responsible for the facility: Dr. Pascal Divanach, Director. Responsible Veterinarian: Giamalaki Eleni. The above-mentioned registered facility is subject to inspection by the responsible Veterinary Directorate regarding the compliance with the provisions of Presidential Decree PD 160/92 (A' 64), 2007/526/EK (L197) Commission Recommendation and PD 184/1996 (A' 137). In any case the authors declare that all relevant ethical safeguards were observed in relation to animal experimentation: in particular each fish was first anaesthetized with clove oil, 100 ppm, for 4 minutes and then painlessly sacrificed.

### 2. Rearing methodologies

#### 2.1 Semi-intensive conditions – Large Volumes

The Italian lots were reared using a semi-intensive methodology known as Large Volumes [47]–[48], used for larval rearing of sea bass (*Dicentrarchus labrax*), gilthead seabream, mullets (*Chelon labrosus*, *Mugil cephalus*), dusky grouper (*Epinephelus marginatus*) [16], [18], [49]–[55]. This method is based on the ecological knowledge of euryhaline finfish larvae, and it is aimed at mimicking the environmental conditions of natural nursery areas, with particular attention paid to water volumes, hydrodynamism, larvae density and prey availability and variety. Circular rearing tanks had a volume of 60 m^3^ (diameter 8 m, water height 1.4 m), in which 3 day post hatching (hereafter dph) larvae were initially stocked at a density of ≤16 larvae/L. The tank's circular shape and surface/depth ratio and the presence of a special radial air-lifter allowed hydrodynamic laminar flow to be generated and prevented the generation of vortices. The air-lifter consists of a box, large about 1/3 the tank radius, containing a linear air diffuser. It establishes differential hydrodynamics inside the tank, stronger in the centre and gradually weaker towards the edges, thus allowing larvae to choose the preferred water current. The presence of superficial skimmers allows oily films to be removed from the water surface.

The tanks were filled with sea water and 2 days before stocking the larvae, unicellular green algal species (*Chlorella minutissima*, *Isochrysis galbana*, *Nannochloropsis suecica*, *N. oculata*: 0.02–0.2×10^6^ cells/cc) and a culture of enriched rotifers (*Brachionus plicatilis* sp., DHA Protein Selco for 8 h) were added; the temperature was around 20°C under natural photoperiod. Oxygen levels were ranging between 5.8 and 7.8 mg/L. After the swim bladder activation phase, the rearing tanks were connected to the external lagoon, where a natural zooplankton assemblage was present. Input water was filtered (200–500 µm) but not sterilized, in order to allow the entry and natural build up of a self-sustaining natural food web based on cultured phyto- and zooplankton, and on wild zooplankton (*Pseudonychocamptus proximus*, *Tisbe holoturiae*, *Nitocra spinipes*, polychaete larvae, bivalves larvae) in the tank. The establishment of a bacterial assemblage increases the self-depuration capacity of the system. This *pabulum* represents a source of natural food which plays an important trophic role, both as energy source and in terms of learning feeding behaviour [48], [55]. Cultured live food (*Brachionus* spp. and *Artemia salina* nauplii and metanauplii) was also supplied to rearing tanks as the main food source for larvae.

Water remained stagnant from 1 to 5 dph, then continuously changed at a 20% daily rate up to day 20 and then gradually increased by up to 100% per day.

#### 2.2 Semi-intensive conditions – Mesocosms

Circular flat-bottomed 40 m^3^ tanks with a water depth of about 2 m were initially stocked with approximately 3–4 yolk-sac gilthead seabream larvae/L at the facilities of the Hellenic Center for Marine Research, Heraklion, Greece. *C. minutissima* was added prior to first feeding and until the end of the period of feeding with rotifers (4–25 dph). Enriched *Artemia* metanauplii were added from 14 up to 50 dph, whereas artificial diet was added after 25 dph. Rotifers were enriched by overnight incubation in Protein DHA Selco (Inve A/S, Belgium) and *Artemia* metanauplii were enriched by overnight incubation in Easy DHA Selco (Inve A/S, Belgium). Prey item concentration was kept to a minimum of 2–3 rotifers/mL and 0.2–0.3 metanauplii/mL. A continuous flow was maintained throughout the rearing period (20% of tank volume/day renewal at the outset, and increasing thereafter). Oil surface layer was disrupted by aeration at four points of the surface area and by the use of an arm placed at the centre of tank which was used to concentrate the oil film in a small area of the tank before it was removed manually. Water temperature was kept at 19±1°C. Rearing in Mesocosms was continued until the fish were 50 days old, when they were moved to rectangular 5 m^3^ tanks.

#### 2.3 Intensive conditions – Italy

Larvae were reared in cylindro-conical 9 m^3^ tanks and stocked at a density of 100 larvae/L. Each tank was equipped with an air lift system similar to that of Large Volumes to improve the hydrodynamics. The water was circulated using a 6 m^3^ biofilter unit. Larvae were fed rotifers from 4 dph up to 30 dph, *Artemia* enriched metanauplii (24–50 dph), whereas artificial diet was added after the 30 dph. Live food was enriched with Easy DHA Selco (Inve A/S, Belgium). The microalgae *Chlorella*, *Isochrisys*, *Nannochloropsis*, and *Tetraselmis* sp. were added to the tanks to support rotifers.

#### 2.4 Intensive conditions – Greece

During the first rearing phase, two cylindroconical 500-L tanks were used supported by a 1 m^3^ biofilter unit. Each tank was stocked to a density of 100 yolk-sac gilthead seabream larvae/L. An air-lift pump inside each unit supplied a continuous movement of water even when the water supply from the biofilter unit was very low [56]. The larvae were fed rotifers (4–30 dph), *Artemia* enriched metanauplii (24–50 dph), whereas artificial diet was added after 30 dph. Live food was enriched as described above in the protocol for Mesocosms units. The microalgae *Chlorella minutissima* was added during the period of feeding with rotifers. After 55 days rearing, about 5,000 fish were transferred to rectangular 5 m^3^ tanks.

### 3. Samples

Specimens were anaesthetized (clove oil: 100 ppm, for 4 minutes), fixed in 10% formalin buffered with phosphate buffer (pH 7.2, 0.15 M) and *in toto* double-stained for cartilage and bone, according to Dingerkus and Uhler [57]. Two different and independent operators measured the standard length (mm) and performed the skeletal anomaly (SD) and meristic count (MC) analyses for each individual. Standard length (SL; mm) was measured from the tip of the snout to the distal edge of the hypural bones, rounded off to the upper 0.5 mm. Observations were performed on both sides of stained samples under a stereomicroscope (Wild, LEITZ). Skeletal anomalies were classified using a dichotomic indicator, where the letter indicates the skeletal element affected and the number the typology of the anomaly (Table 2). The anatomical terminology is according to [58] and [4], with the exception of terminology for caudal fin structures, which is according to [59].

**Table 2:** List of considered anomalies.

	Code	Description
*Region*	A	Cephalic vertebrae (carrying epipleural ribs)
	B	Pre-haemal vertebrae (carrying epipleural and pleural ribs and open haemal arch, without haemal spine)
	C	Haemal vertebrae (with haemal arch closed by haemal spine)
	D	Caudal vertebrae (with haemal and neural arches closed by modified spines)
	E	Pectoral fin
	F	Anal fin
	G	Caudal fin
	H	Dorsal spines
	I	Dorsal soft rays
	L	Pelvic fin
*Anomalies*	*1*	*Kyphosis*
	*2*	*Lordosis*
	*3*	*Partial vertebral fusion*
	3*	*Total vertebral body fusion*
	*4*	*Vertebral anomaly (shape anomaly, ossification ridges, marked reduction in length or elongation, intervertebral bony plate)*
	5	Anomalous neural arch and/or spine
	5*	Supernumerary neural elements/absence of neural elements
	6	Anomalous haemal arch and/or spine
	6*	Supernumerary haemal elements/absence of haemal elements
	7	Anomalous rib
	7*	Supernumerary pleural rib
	8	Anomalous pterygiophores (anomalous, absent, fused, supernumerary)
	9	Anomalous hypural (anomalous, absent, fused, supernumerary)
	9*	Anomalous or broken parahypural or fused with hypural/haemaspine
	10	Anomalous epural (anomalous, absent, fused, supernumerary)
	11	Anomalous ray (anomalous, absent, fused, supernumerary)
	*12*	*Swim bladder anomaly*
	*13*	*Presence of calculi in the urinary ducts*
	*14*	*Anomalous maxillary and/or pre-maxillary*
	*15*	*Anomalous dentary*
	*16*	*Other cephalic deformities (glossohyal, neurocranium, ..)*
	*17L/R*	*Anomalous left/right opercular plate*
	17*L/R	*Anomalous, absent, fused branchiostegal ray*
	18	Predorsal bones anomalies
	19	Hypural with decalcifications
	20	Decalcified pterygophore
	21	Anomalous epipleural ribs
	22	Anomalous dorsal ribs
	23	Anomalous pleural ribs
	24 L/R	Decalcified left/right opercular plate
	25	Epural with decalcifications
	26	Supernumerary bone
	27	Decalcified urostyle
	28	Decalcified vertebrae
	29	Anomalous postcleithrum
	*S*	*Scoliosis*
	Cl L/R	Anomalous left/right cleithrum
	Cor L/R	Anomalous left/right coracoid

Italics highlight severe anomalies, defined as those that affect the external shape of the fish body.

The following derived variables were computed for each lot:

1. relative frequency (%) of individuals with at least one anomaly;
2. number of anomaly typologies observed;
3. average anomalies load (number of total anomalies/number of malformed individuals);
4. relative frequency (%) of individuals with at least one severe anomaly;
5. ratio (%) of observed severe anomalies on the total number of observed anomalies;
6. severe anomalies load (number of severe anomalies/number of individuals with severe anomalies);
7. frequency (%) of each anomaly typology, with respect to the total number of anomalies observed in each lot.

Severe anomalies refer to those typologies that affect the external shape of the fish.

Counts of the following meristic characters were carried out: total vertebrae (including urostyle), anal rays, first and second dorsal rays (divided into spines and soft rays, respectively), principal caudal fin rays (divided into upper caudal rays and lower caudal rays), pectoral and pelvic fin rays (left and right side), the inner supports of fins (pterygiophores, hypurals, epurals, radials) and predorsal bones. Data referring to the groups 1 and 2 were collected in 1997, when only the total number of vertebrae (including the urostyle) and fin rays were taken into account in meristic count.

The analysis was carried out on the basis of certain assumptions: i) non-completely fused bone elements were counted as distinct elements in meristic counts; ii) supernumerary bones with a normal morphology were not considered as anomalies but included as meristic count variations; conversely, anomalous supernumerary elements were included among anomalies; iii) only the clearly and unquestionably identifiable variations in shape were considered as skeletal anomalies: if any doubts arose, then the shape variation was not considered anomalous; iv) misalignments of vertebrae were considered as lordosis and/or kyphosis only if the vertebral bodies involved were deformed.

Data obtained from meristic counts were compared with those obtained from 5 other conspecific wild juvenile lots (277 individuals; see Table 3) drawn from our Lab's historical database and used here as quality reference standard (*wild-like* phenotype, according to [48]).

**Table 3:** Characteristics of wild gilthead seabream lots belonging to the historical dataset.

Lot code	Origin	n	Mean SL	Range
**WIIT01**	Adriatic sea (off Italian coast)	72	19.9	9.5–49
**WIIT02**	Adriatic sea (off Italian coast)	41	38.1	25–43
**WIIT03**	Adriatic sea (off Italian coast)	60	58.1	52–70
**WIIT04**	Adriatic sea (off Italian coast)	16	20.0	17–22
**WITU01**	Aegean sea (off Turkish coast)	88	19.8	11.5–44
***Total***		***277***		***9.5–70***

*n* = number of individuals; *SL* = standard length, in mm; *Range* = observed minimum and maximum standard length, for each lot, in mm.

Data obtained from the analysis of skeletal anomalies in reared groups were transformed into a binary matrix (hereafter named BM: presence of each skeletal anomaly typology = 1; absence = 0). Another matrix (FM) was built by calculating the frequency of each typology, in each lot, from the BM. Both BM and FM were then subjected to Correspondence Analysis (CA) [60] in order to visualize the relationships among lots and the role that each anomaly plays in defining the characteristics of different lots. In order to correctly represent the frequency of specimens without abnormalities during CA vector normalization, a binary variable (ABS) was used to distinguish between those individuals expressing at least one skeletal anomaly and individuals without any anomalies. A unit value (i.e. true) was used for specimens with no anomalies and a null value for specimens with at least one anomaly. All the anomaly typologies indicated with an asterisk in the code (see Table 2) were merged in the FM matrix with the main typology: i.e., 3* was merged with 3; 17dx, 17sx, 17*dx and 17*sx were merged into a single typology, denoted as 17 (deformed opercular plate, including branchiostegal ray deformation).

The relative frequencies of individuals affected by each anomaly typology in the lots of each Group were presented with tables and radar plots.

In order to test the significance of the differences in frequencies of individuals affected by each anomaly typology among the different lots of each Group, a simple matching similarity matrix [61] was computed and then subjected to two-way NPMANOVA (Past version 2.14, available at: www.nhm.uio.no/norlex/past/download.html [62]).

## Results

### 1. Meristic counts

The results of meristic counts are shown in tables 4 and 5, in which only the range of variation in comparison with wild lots (Table 4) and median and ranges values are reported for the sake of comparison among sister lots (Table 5).

**Table 4:** Results of meristic counts: comparison of ranges observed in reared and wild lots.

			Caudal fin				Anal fin		Dorsal fin					Pectoral fin				Pelvic fin	
	Lots	Vertebrae	Hypurals	Epurals	Upper rays	Lower rays	Pterygiophores	Rays	Predorsal bones	Hard rays pterygiophores	Hard rays	Soft rays pterygiophores	Soft rays	Left side rays	Right side rays	Left side radials	Right side radials	Left side rays	Right side rays
**WILD**	**WIIT01** *	24	n.c.	n.c.	9	7–8	n.c.	13–16	n.c.	n.c.	10–11	n.c.	12–13	13–16	n.c.	n.c.	n.c.	n.c.	n.c.
	**WIIT02**	24	4–5	3–4	9	8	11–13	14–16	3	9–11	11–12	11–14	13–15	15–17	15–17	4	4	6–7	6–7
	**WIIT03** *	24–25	3–5	3	8–9	8	11–13	14–16	3	9	11	12–14	12–15	15–17	n.c.	4	n.c.	n.c.	n.c.
	**WIIT04**	24	5	3–4	9	8	13–14	15–16	3	10	11	13–14	14–15	15–16	15–16	4	4	6	6
	**WITU01** *	24	n.c.	n.c.	9	7–8	n.c.	13–15	n.c.	n.c.	11	n.c.	12–14	15–17	n.c.	n.c.	n.c.	n.c.	n.c.
**Group 1**	**INIT06**	**23–24**	n.c.	n.c.	8–9	**8–10**	n.c.	13–16	n.c.	n.c.	**9–11**	n.c.	**11–15**	15–17	n.c.	n.c.	n.c.	n.c.	n.c.
	**LVIT01**	24–25	n.c.	n.c.	8–9	8	n.c.	14–15	n.c.	n.c.	10–12	n.c.	12–13	15–16	n.c.	n.c.	n.c.	n.c.	n.c.
**Group 2**	**INIT07**	**23–25**	n.c.	n.c.	8–9	**8–9**	n.c.	13–16	n.c.	n.c.	**9–14**	n.c.	**11–14**	**7–16**	n.c.	n.c.	n.c.	n.c.	n.c.
	**LVIT02**	24–25	n.c.	n.c.	9	**8–9**	n.c.	14–16	n.c.	n.c.	**7–11**	n.c.	12–15	13–16	n.c.	n.c.	n.c.	n.c.	n.c.
**Group 3**	**INIT19**	**23–25**	**5–6**	**2–4**	**8–10**	**8–9**	**9–13**	**11–16**	**2–4**	**7–10**	**9–11**	**11–15**	**12–16**	14–16	**0–17**	4	**0–4**	**0–7**	**0–7**
	**INIT18**	**23–25**	4–5	**2–4**	9	**8–9**	11–13	**14–17**	**2–3**	**7–9**	**9–11**	**11–15**	**12–16**	14–16	**14–16**	4	**3–4**	6–7	6–7
	**LVIT04**	24	5	**2–3**	8–9	**8–9**	12–13	14–16	**2–3**	**7–9**	10–11	12–14	13–15	14–16	**14–15**	4	4	6–7	6–7
	**LVIT05**	24–25	4–5	**2–4**	9	**8–9**	11–13	14–16	**2–3**	**7–9**	**9–11**	**12–15**	13–15	14–16	**14–16**	4	**3–6**	6	6
**Group 4**	**INGR01**	**24–26**	**4–7**	**2–6**	**6–10**	**5–9**	**13–15**	14–16	**2–3**	10–11	11–12	11–14	12–14	14–16	**14–16**	4	4	**5–6**	**5–6**
	**MEGR04**	24–25	**4–7**	**2–6**	**6–9**	**5–9**	**13–15**	**15–17**	**3–4**	10–11	11	12–14	13–15	14–15	**14–16**	4	4	6	6

Bold values: range outside the wild one; *n.c.* = data not collected;

* = observation carried out only on the left side of the body.

**Table 5:** Results of meristic counts: comparison of observed median and range values between sibling lots.

				CAUDAL FIN				PECTORAL FIN				PELVIC FIN		ANAL FIN		DORSAL FIN				
	Lots	Value	Vertebrae	Epurals	Hypurals	Upper rays	Lower rays	Left side rays	Right side rays	Left side radials	Right side radials	Left side rays	Right side rays	Pterygiophores	Rays	Predorsal bones	Hard rays pterygiophores	Soft rays pterygiophores	Hard rays	Soft rays
**Group 1**	**INIT06**	*Median*	24	n.c.	n.c.	9	8	**16**	n.c.	n.c.	n.c.	n.c.	n.c.	n.c.	14	n.c.	n.c.	n.c.	**10**	13
		*Min*	**23**	n.c.	n.c.	8	8	15	n.c.	n.c.	n.c.	n.c.	n.c.	n.c.	**13**	n.c.	n.c.	n.c.	**9**	**11**
		*Max*	**24**	n.c.	n.c.	9	**10**	**17**	n.c.	n.c.	n.c.	n.c.	n.c.	n.c.	**16**	n.c.	n.c.	n.c.	**11**	**15**
	**LVIT01**	*Median*	24	n.c.	n.c.	9	8	**15**	n.c.	n.c.	n.c.	n.c.	n.c.	n.c.	14	n.c.	n.c.	n.c.	**11**	13
		*Min*	**24**	n.c.	n.c.	8	8	15	n.c.	n.c.	n.c.	n.c.	n.c.	n.c.	**14**	n.c.	n.c.	n.c.	**10**	**12**
		*Max*	**25**	n.c.	n.c.	9	**8**	**16**	n.c.	n.c.	n.c.	n.c.	n.c.	n.c.	**15**	n.c.	n.c.	n.c.	**12**	**13**
**Group 2**	**INIT07**	*Median*	24	n.c.	n.c.	9	8	15	n.c.	n.c.	n.c.	n.c.	n.c.	n.c.	14	n.c.	n.c.	n.c.	11	13
		*Min*	**23**	n.c.	n.c.	**8**	8	**7**	n.c.	n.c.	n.c.	n.c.	n.c.	n.c.	**13**	n.c.	n.c.	n.c.	**9**	**11**
		*Max*	25	n.c.	n.c.	9	9	16	n.c.	n.c.	n.c.	n.c.	n.c.	n.c.	16	n.c.	n.c.	n.c.	**14**	**14**
	**LVIT02**	*Median*	24	n.c.	n.c.	9	8	15	n.c.	n.c.	n.c.	n.c.	n.c.	n.c.	14	n.c.	n.c.	n.c.	11	13
		*Min*	**24**	n.c.	n.c.	**9**	8	**13**	n.c.	n.c.	n.c.	n.c.	n.c.	n.c.	**14**	n.c.	n.c.	n.c.	**7**	**12**
		*Max*	25	n.c.	n.c.	9	9	16	n.c.	n.c.	n.c.	n.c.	n.c.	n.c.	16	n.c.	n.c.	n.c.	**11**	**15**
**Group 3**	**INIT19**	*Median*	24	3	5	9	8	15	15	4	4	6	6	12	15	3	8	13	10	14
		*Min*	**23**	2	5	8	8	14	**0**	4	**0**	**0**	**0**	**9**	**11**	2	7	**11**	**9**	**12**
		*Max*	**25**	**4**	**6**	**10**	9	16	**17**	4	4	7	7	13	16	**4**	**10**	**15**	11	**16**
	**LVIT04**	*Median*	24	3	5	9	8	15	15	4	4	6	6	12	15	3	8	13	10	14
		*Min*	**24**	2	5	8	8	14	14	4	**4**	6	**6**	**12**	**14**	2	7	**12**	**10**	**13**
		*Max*	**24**	**3**	**5**	**9**	9	16	**15**	4	4	7	7	13	16	**3**	**9**	**14**	11	**15**
	**INIT18**	*Median*	24	2	5	9	8	15	15	4	4	6	6	12	15	3	**9**	13	**11**	14
		*Min*	**23**	2	4	9	8	14	14	4	3	6	6	11	14	2	7	**11**	9	**12**
		*Max*	25	4	5	9	9	16	16	4	**4**	**7**	**7**	13	**17**	3	9	15	11	**16**
	**LVIT05**	*Median*	24	2	5	9	8	15	15	4	4	6	6	12	15	3	**8**	13	**10**	14
		*Min*	**24**	2	4	9	8	14	14	4	3	6	6	11	14	2	7	**12**	9	**13**
		*Max*	25	4	5	9	9	16	16	4	**6**	**6**	**6**	13	**16**	3	9	15	11	**15**
**Group 4**	**INGR01**	*Median*	25	6	4	9	8	**14**	15	4	4	6	6	**13**	15	3	10	13	11	14
		*Min*	24	4	2	6	5	14	14	4	4	**5**	**5**	13	**14**	**2**	10	**11**	11	**12**
		*Max*	**26**	7	6	**10**	9	**16**	16	4	4	6	6	15	**16**	**3**	11	14	**12**	**14**
	**MEGR04**	*Median*	25	6	4	9	8	**15**	15	4	4	6	6	**14**	15	3	10	13	11	14
		*Min*	24	4	2	6	5	14	14	4	4	**6**	**6**	13	**15**	**3**	10	**12**	11	**13**
		*Max*	**25**	7	6	**9**	9	**15**	16	4	4	6	6	15	**17**	**4**	11	14	**11**	**15**

Bold values highlight differences with siblings of the same age; *n.c.* = data not collected.

Wild counts among the different wild lots were very similar. The lot with the widest variation range was that with the largest specimens (TL ranging from 52 to 70 mm: lot WIIT03); however, this variation in the majority of characters (e.g. higher number of vertebrae, low number of hypurals and caudal upper rays) was due to a small number of individuals. Only the presence of 4 hypurals is extended to a larger number of individuals (27% of WIIT03 juveniles).

The numbers of vertebrae and caudal upper rays are those best conserved in all the other wild lots, whilst pectoral rays and elements of the soft portion of dorsal fins are the most variable.

The intensively reared lots all showed a higher number of meristic counts outside the wild range than semi-intensive ones, albeit limited to only a few individuals or to certain meristic counts in some cases. Most of the altered meristic counts involved normally shaped elements: i.e., 29.1% of individuals in INGR01 lot displayed a different number of caudal rays, whereas only 1.5% showed both an anomalous caudal ray count and anomalous rays.

The number of vertebrae in intensively reared juveniles was a very interesting character, ranging from 23 (4% of the specimens) to 26 (1%). However, in 72% of the specimens 24 vertebrae were counted, and 25 in the remaining 23%. The semi-intensively reared juveniles showed a vertebral number ranging between 24 and 25 only (as in the wild). A more detailed description of the characters which varied in a substantial number of individuals or that showed large differences between the sister lots is reported below for each group.

#### Group 1

The Large Volumes lot in this group (LVIT01) showed no differences with respect to the wild meristic count range, while the intensive sister one (INIT06) showed differences in the number of vertebrae, caudal lower rays and dorsal hard rays (Table 4). In addition, differences were observed between the two lots in the median values for certain characters (e.g. left pectoral rays and dorsal hard rays) and in the number of frequency classes (e.g. anal rays) (Table 5).

#### Group 2

The Large Volumes lot (LVIT02) showed substantial differences with respect to the wild meristic count range in the number of caudal lower rays, and the intensive lot (INIT07) in the number of caudal lower rays (Table 4).

As far as the comparison among sister lots was concerned, in this group the distribution of frequency classes showed similar trends in the two lots, with the exception of the caudal lower and dorsal hard rays: the intensive lot showed a higher frequency (34.1% *vs* 13.1%) of individuals with a larger number of caudal lower rays (9 instead of 8), and a larger number of frequency classes in the number of dorsal hard rays (9, 10, 11 and 14 *vs* 3, 7, 10 and 11) than the sister lot reared in semi-intensive conditions (Table 5).

#### Group 3

Semi-intensive lots showed a generally lower variability than intensive ones with respect to wild counts. In particular, differences among LVIT04 and wild lots were found in the number of epurals, caudal lower rays, pre-dorsal bones, and dorsal hard ray pterygiophores. LVIT05, in their turn, showed considerable differences vis-à-vis the wild lots in the number of epurals, caudal lower rays, predorsal bones and dorsal hard pterygiophores. The intensive lots showed a higher number of individuals with meristic counts outside the range observed in wild samples. The majority of the differences versus wild counts were found in the number of the epurals, predorsal bones and first dorsal pterygiophores in INIT18, and in the number of caudal lower rays, predorsal and the first dorsal pterygiophores in INIT19.

As far as comparison between siblings is concerned, the semi-intensive siblings showed an overall lower variability than the corresponding intensive ones of the same age. In particular, two median values were found to be different in the 63–64 dph lots: the first dorsal fin rays and the pterygiophores (Table 5).

Some differences were found in lots produced by the same rearing methodology but sampled at different ages.

#### Group 4

The intensive lot showed a higher number of characters (9) found to lie outside the wild range than the Mesocosm lot (7 characters). The number of vertebrae and pelvic rays change with respect to the wild one only in INGR01 and the number of anal rays only differ in MEGR04. The observed variation of the number of pelvic rays involved always only one of the two fins, and not on the same side, in the two INGR01 individuals affected. The intensive lots showed some individuals (3%) with 26 vertebrae (*vs* 0% in MEGR04), 79% with 25 (*vs* 67% in MEGR04) and 18% (*vs* 33% in MEGR04) with 24 vertebrae (Table 4).

Some differences in the median values between the two lots were found, such as in the number of anal pterygiophores and left pectoral rays (Table 5).

### 2. Skeletal anomalies

**Figure 1:**
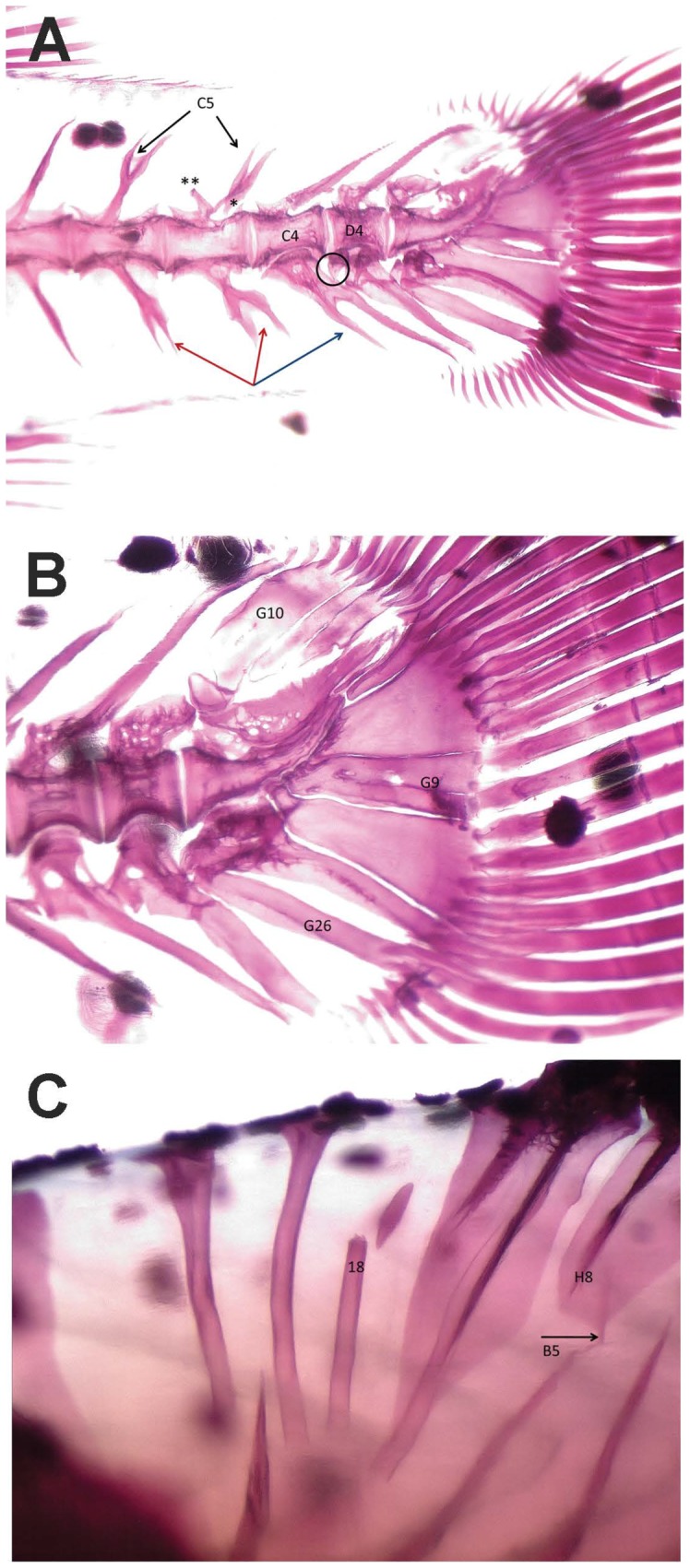
Example of skeletal anomalies in gilthead seabream. A – Vertebrae anomalies: deformed bodies of haemal (C4) and caudal (D4) vertebrae; forked neural spines (C5; black arrows) and detachment (*) and anomalous osseous bulge (**) in neural arch in haemal vertebrae (C5); forked haemal spines of two haemal (C6; red arrows) and one caudal vertebra (D6; blue arrow); supernumerary, defective, haemal arch of caudal vertebra (D6; black circle); heterotopic, mineralized skeletal element in the caudal fin (G26). B - Anomalies affecting caudal fin in gilthead seabream: partially fused epurals (G10); partially fused 2^nd^ and 3^rd^ hypurals (G9); heterotopic mineralized element in the caudal region (G26). C – Heterotopic neural spine of a pre-haemal vertebra (B5; arrow); fractured predorsal bone (18); shorter pterygiophore (H8).

In Table 6, the general data obtained from the skeletal anomalies analysis are reported; in Fig. 1 some observed skeletal anomalies are shown. Almost always, reared fish showed higher rates of anomalous specimens than wild ones: the total frequency (%) of individuals affected by at least one anomaly ranged from 87% to 100%, whilst in wild lots this value ranged from 43.2 to 100% of juveniles. It should be stressed that 100% of deformed wild individuals was observed only in lot WIIT04, which was composed only of 16 individuals. Deformed individuals exhibited an average anomalies charge ranging from 3 to 9 anomalies/individual in reared lots, and from 1 to 6 (the latter value was found exclusively in lot WIIT04) in the wild ones. WIIT04 juveniles, on the other hand, showed no severe anomalies, while the other wild lots had frequencies varying from 1.7 to 4.5% of individuals, with 1–3 severe anomalies per individual. In reared lots, a frequency varying from the 8.6 up to the 74.5% of individuals was found to be affected by 1–5 severe anomalies per individual.

**Table 6:** Results from skeletal anomalies analysis: general data (see text for further details).

	Code	N. individuals	Frequency (%) of malformed individuals	Average anomalies load	Frequency of individuals with at least one severe anomaly (%)	Severe anomalies load	Frequency (%) of severe anomalies/total
**Group 1**	**INIT06**	55	**100**	**8.6**	**74.5**	1.8	16
	**LVIT01**	66	**100**	3.6	54.5	1.3	19.5
**Group 2**	**INIT07**	123	95.9	3.8	52.8	1.6	**23.2**
	**LVIT02**	122	98.4	2.7	48.4	1.2	21.2
**Group3**	**INIT19**	105	96.2	5.8	47.6	1.6	13.4
	**LVIT04**	40	92.5	3.9	12.5	**6.0**	**16.5**
	**INIT18**	105	86.7	3.5	22.9	1.8	13.7
	**LVIT05**	105	96.2	2.8	8.6	1.3	4.3
**Group 4**	**INGR01**	134	95.5	4.2	28.4	1.5	10.5
	**MEGR04**	126	93.6	4.3	27	1.3	9.1
**Wild**	**WIIT01**	72	54.8	1.4	4.2	2.3	21.2
	**WIIT02**	41	43.9	**1.2**	2.4	1	4.8
	**WIIT03**	60	55	1.6	1.7	3	5.8
	**WIIT04**	16	**100**	6.3	**0**	**0**	**0**
	**WITU01**	88	**43.2**	1.6	4.5	1.5	9.7

Bold type highlights the lowest and highest values.

The data referring to relative frequencies (%) of each anomaly typology on the total observed anomalies in each lot (Table 7), and the frequency (%) of individuals affected by each anomaly typology, per lot (Table 8), evidenced some general trends, as follows:

**Table 7:** Relative frequencies (%) of each anomaly typology, in each lot.

	Group 1		Group 2		Group 3				Group4	
	INIT06	LVIT01	INIT07	LVIT02	INIT19	LVIT04	INIT18	LVIT05	INGR01	MEGR04
***A1***		*0.4*	*0.2*	*0.6*						
***A2***							*0.3*			
***A3***						*1.4*				
***A4***						*1.4*				
**A5**	0.6	0.8	3.1	1.8	**4.9**	2.8	0.9	0.3	0.2	0.2
A5*							0.3	0.3		
***B1***					*0.3*	*0.7*				
***B2***			*0.2*		*0.3*	*0.7*				
***B3***					*0.5*	***5.5***	*0.3*			
***B4***		*0.8*	*0.9*		*0.3*	*3.4*				
**B5**		0.4	2.0	0.6	2.7	**4.8**				
***C1***	*0.6*	*0.8*			*0.2*		*0.9*	*1.8*		
***C2***					*0.2*					
***C3***					*0.2*	*0.7*	*0.3*			*0.4*
C3*									***0.2***	
***C4***	*1.3*	*1.7*	*1.1*	*0.9*	*0.7*	*0.7*	*1.2*	*0.3*	*0.7*	*1.4*
**C5**	**4.4**	1.7	3.5	0.3	**7.8**	**5.5**	**6.9**	3.5	1.7	**7.9**
C5*					0.3			**6.7**	0.2	
**C6**	**4.0**	2.1	**4.2**	**8.3**	**5.2**	**6.2**	**4.4**	**4.6**	2.8	**4.8**
C6*							0.3	0.3	0.2	0.2
***D1***					*0.3*					
***D2***										*0.2*
***D3***	*2.3*	*0.4*	***0.4***		*3.6*	***0.7***	*1.9*		*1.5*	*0.6*
D3*									*0.4*	*0.6*
***D4***	***5.7***	***6.8***	***4.2***	*2.8*	*3.6*	*1.4*	***5.3***	*2.1*	*3.9*	*2.9*
**D5**	**8.0**	0.8	3.5	0.6	**7.3**	2.1	**6.2**	1.4	0.7	2.8
D5*					1.9	0.7	0.9	0.3	1.3	2.8
**D6**	**10.7**	**10.6**	**7.7**	1.5	**5.2**	**5.5**	3.7	**12.1**	**4.7**	2.8
D6*					**5.9**	**17.2**	**9.4**	**19.9**	3.9	2.8
**E8L**					1.0		0.6		0.6	0.2
**E8R**					1.7		0.6	0.3	1.1	0.2
**E11L**	2.9							0.3	**6.4**	**8.9**
**E11R**					0.2	1.4			2.4	**6.7**
**F8**	**6.1**	0.8	1.3	0.9	2.7	3.4	2.8	3.5	2.2	1.8
**F11**	0.4	3.8	0.7	1.2	2.7	0.7	2.8	0.3	0.9	0.2
**G9**	**10.9**	**33.0**	**19.9**	**34.8**	**5.8**	2.8	**15.3**	**14.2**	**30.0**	**18.1**
G9*									2.8	**4.8**
**G10**	**10.3**	**14.0**	**15.0**	**7.4**	**8.0**	**8.3**	**11.9**	**13.1**	2.2	**11.3**
**G11**	**6.3**		**5.3**	**5.8**	1.2	0.7	1.6		2.4	2.8
**G26**									**6.9**	3.2
**H8**	**12.2**	**4.7**	0.7	2.5	**9.7**	**11.0**	**6.6**	**8.2**	1.9	1.8
**H11**	1.3	1.3	**8.0**	**9.2**	1.9				**5.4**	1.6
**I8**	0.8	0.8	1.5	1.5	3.2	2.1	1.6	1.4	2.8	1.8
**I11**	**4.8**	**5.1**	0.2	2.1	0.7		0.3	0.3	2.6	1.8
**L8R**					0.2		0.3			
**L11L**					0.2	0.7			1.5	0.2
**L11R**					0.2				0.4	
***12***	*0.2*			***0.3***						
***14***			***0.7***						*2.8*	*2.9*
***15***	*3.4*	***4.2***	***8.4***	***14.1***	*0.3*					*0.4*
***16***	*0.2*	***4.2***	*1.1*	*1.2*						
***17L***	*2.3*	*0.4*	***6.0***	*1.2*	*1.5*		***2.2***		*0.6*	
***17R***					*1.4*		*1.2*		*0.4*	
17*L					*0.3*	*0.7*				
17*R					*0.2*	*0.7*				
**18**					3.9	**4.8**	**8.4**	**4.3**	0.9	1.2
**19**					0.2	1.4	0.3			
**20**					0.3					
**25**										0.2
**ClL**					0.7					
**ClR**					0.5					

When the observed value is 0, the cell has been left empty to make reading easier.

Codes in italics highlight severe anomalies; bold digits highlight the highest values of frequency.

**Table 8:** Relative frequencies (%) of individuals affected by each anomaly typology, in each lot.

	Group 1		Group 2		Group 3				Group 4	
	INIT06	LVIT01	INIT07	LVIT02	INIT19	LVIT04	INIT18	LVIT05	INGR01	MEGR04
***A1***		*1.5*	*0.8*	*1.6*						
***A2***							*0.9*			
***A3***						***5.0***				
***A4***						***5.0***				
**A5**	**5.4**	3.3	**11.4**	**4.9**	**25.7**	**7.5**	2.9	0.9	0.7	0.8
A5*							0.9	0.9		
***B1***					*1.9*	*2.5*				
***B2***			*0.8*		*1.9*	*2.5*				
***B3***					*2.9*	*2.5*	*0.9*			
***B4***		*1.5*	*2.4*		*1.9*	*2.5*				
**B5**		1.5	**4.9**	1.6	**7.6**	**5.0**				
***C1***	***5.4***	*3.3*			*0.9*		*2.9*	***4.8***		
***C2***					*0.9*					
***C3***					*0.9*	*2.5*	*0.9*			*1.6*
C3*									*0.7*	
***C4***	***5.4***	***4.5***	*2.4*	*2.5*	*2.9*	*2.5*	*3.9*	*0.9*	*3.0*	***4.8***
**C5**	**25.4**	**6.7**	1.6	0.8	**35.2**	**15.0**	**16.2**	**7.6**	**6.7**	**23.2**
C5*					1.9			**14.3**	0.7	
**C6**	**30.0**	**7.6**	**13.8**	**18.8**	**27.6**	2.0	**13.3**	**11.4**	1.4	**17.5**
C6*							0.9	0.9	0.7	0.8
***D1***					*1.9*					
***D2***										*0.8*
***D3***	*2.0*	*1.5*	*1.6*		***19.5***	*2.5*	***5.7***		***6.0***	*2.4*
D3*									*1.5*	*2.4*
***D4***	***38.2***	***22.7***	***13.8***	***7.4***	***15.2***	*2.5*	***8.6***	*3.9*	***8.3***	***7.9***
**D5**	**52.7**	3.3	**11.4**	1.6	**35.2**	**7.5**	**18.9**	3.9	3.0	**7.9**
D5*					1.5	2.5	2.9	0.9	**5.2**	**7.9**
**D6**	**72.7**	**31.8**	**23.6**	**5.0**	**23.9**	**17.5**	1.5	**32.4**	**17.9**	**7.9**
D6*					**33.3**	**6.0**	**28.6**	**53.3**	**15.7**	**7.9**
**E8L**					**5.7**		1.9		2.2	0.8
**E8R**					**9.5**		1.9	0.9	3.7	0.8
**E11L**	**10.9**							0.9	**16.4**	**21.4**
**E11R**					0.9	2.5			**8.3**	**15.9**
**F8**	**36.4**	3.3	**4.9**	2.5	1.5	1.0	**8.6**	**8.6**	**7.5**	**5.6**
**F11**	3.6	**4.5**	2.4	3.3	**15.2**	2.5	**8.6**	0.9	3.7	0.8
**G9**	**70.0**	**87.9**	**56.9**	**69.7**	**32.4**	1.0	**44.8**	**34.3**	**76.1**	**57.1**
G9*									**11.2**	**19.5**
**G10**	**83.6**	**5.0**	**5.5**	**18.8**	**42.9**	**27.5**	**36.2**	**35.2**	**7.5**	**37.3**
**G11**	2.0		**13.8**	**5.7**	3.9	2.5	**4.8**		**9.0**	**9.5**
**G26**									**26.9**	**12.7**
**H8**	**63.6**	**13.6**	2.4	**5.0**	**45.7**	**37.5**	**19.5**	2.0	**5.2**	**5.6**
**H11**	2.0	**4.5**	**18.7**	**17.2**	**9.5**				**14.9**	**6.3**
**I8**	3.6	3.3	**5.7**	2.5	**16.2**	**7.7**	**4.8**	2.9	**5.2**	**4.0**
**I11**	2.0	**10.0**	0.8	2.5	3.9		0.9	0.9	**8.3**	**6.3**
**L8R**					0.9		0.9			
**L11L**					0.9	2.5			**5.2**	0.8
**L11R**					0.9				0.7	
***12***	*1.8*			*0.8*						
***14***			*2.4*						***11.2***	***11.1***
***15***	***30.0***	***15.1***	***30.9***	***37.7***	*1.9*					*1.6*
***16***	*1.8*	***15.1***	***4.6***	*3.3*						
***17L***	***16.4***	*1.5*	***17.9***	*3.3*	***8.6***		***6.7***		*2.2*	
***17R***					***7.6***		*3.9*		*1.5*	
17*L					*1.9*	***2.5***				
17*R					*0.9*	***2.5***				
**18**					**21.9**	**17.5**	**25.7**	**11.4**	3.7	**4.0**
**19**					0.9	**5.0**	0.9			
**20**					1.9					
**25**										0.8
**ClL**					3.9					
**ClR**					2.9					

When the observed value is 0, the cell has been left empty to make reading easier. Codes in italics highlight severe anomalies; bold digits highlight the highest values of frequency.

- the frequency of some anomaly typologies (i.e. D3, E8sx, E8dx, I8, 17sx and 17dx) diminished in all the siblings reared in both the tested semi-intensive conditions;
- no anomaly typology different from those observed in intensive fish was present in any of the siblings reared in both the semi-intensive conditions;
- the occurrences of anomalies affecting neural arches of cranial, haemal and caudal vertebrae (i.e. A5, C5, D5, D5*) diminished in all sister lots reared in Large Volumes but not in those reared in Mesocosm;
- a series of anomalies (i.e. D4, G10 and G11) showed peculiar trends: the frequencies of individuals affected by anomalies affecting caudal vertebrae bodies (D4) were lower in semi-intensive conditions than in intensively reared sister lots (Table 8), but the incidence of such anomalies versus the total anomalies observed in each lot (Table 7) increased in LVIT01, whereas in the other lots it diminished. This means that a reduced number of LVIT01 individuals showed a higher number of deformed caudal vertebrae centra than in INIT06. The occurrence of anomalies affecting epurals (G10) and caudal principal rays (G11) was lower in Large Volumes individuals, but higher in the Mesocosm lot, with respect to the corresponding intensively reared sister lot; further, the trend of the relative incidence of these two anomalies on the total of anomalies detected in each lot did not follow the descending trend observed in semi-intensive lots. This means that in all the Large Volumes lots fewer individuals were found to be affected by G10/G11 than in intensively reared siblings, but in some of these lots these individuals showed a greater number of deformed epurals and caudal rays;
- no clear differences in the most affected body region were observed among sister lots, with the sole exception of Group 3 where (slight) differences were found only between the two older lots: in LVIT05 the most affected region were the caudal vertebrae (35.8%), while in INIT18 it was the caudal fin (29.1%).

All the other observed differences involved very few individuals (1–2).

In order to determine whether the anomalous counts were due to anomalies (fusions, e.g.) and whether some skeletal elements were influenced more than the others by the rearing conditions, the frequencies of individuals with meristic counts differing from the wild ones, the frequencies of individuals with at least one skeletal anomaly, and the frequencies of individuals carrying both anomalous meristic counts and skeletal anomalies affecting meristic characters were calculated, and data grouped as a function of the mode of development of the affected skeletal elements (Tabs. 9, 10 and 11). The analysis of the data grouped according to the ossification typologies of skeletal elements affected by anomalies evidenced that only anomalies in shape and number (altered meristic counts) of intramembranous bones are influenced by the rearing conditions applied, as evidenced by the lower frequencies of anomalous numbers and shapes of intramembranous bones in most of the semi-intensive lots (Tab 9). In bones with a cartilaginous precursor, the differences in anomalies between intensive and semi-intensive rearing conditions followed less unambiguous and sometimes opposite trends (Tab 10).

**Table 9:** Frequencies (%) of individuals with altered counts, individuals with deformed shape, and individuals with both altered counts and deformed shapes of bones underwent indirect ossification (i.e., endo- or perichondral ossification), per lot.

		Group 1		Group 2		Group 3				Group 4	
		INIT06	LVIT01	INIT07	LVIT02	INIT19	LVIT04	INIT18	LVIT05	INGR01	MEGR04
**N.**		55	66	123	121	105	40	105	105	134	126
**Anomalous number of hypurals**		n.c.	n.c.	n.c.	n.c.	0.95				3.7	4.0
**Anomalous number and shape of hypurals** (G9, G9*, G26)		n.c.	n.c.	n.c.	n.c.					2.2	4.0
**Anomalous shape of hypurals** 1 (G9, G9*, G26)		69.1	87.9	56.9	70.2	32.4	15.0	44.8	34.3	84.3	69.8
**Anomalous number of epurals**		n.c.	n.c.	n.c.	n.c.	1.9	50.0	62.9	50.5	21.6	42.9
**Anomalous number and shape of epurals** (G10)		n.c.	n.c.	n.c.	n.c.		12.5	21.9	14.3		15.1
**Anomalous shape of epurals** (G10)		83.6	40.9	50.4	18.8	42.9	27.5	36.2	35.2	7.5	37.3
**Anomalous shape of vertebral arches** (5, 5*, 6, 6*, 7, 7*)		83.6	42.4	44.7	27.3	85.7	77.5	63.8	84.8	43.3	35.7
**Anomalous number of fin pterygiophores**	**Pectoral (Radials)**	n.c.	n.c.	n.c.	n.c.	0.9		0.9	1.9		
	**Dorsal**	n.c.	n.c.	n.c.	n.c.	81.9	80.0	40.0	67.6		
	**Anal**	n.c.	n.c.	n.c.	n.c.	3.8				1.5	2.4
**Anomalous number and shape of fin pterygiophores** (8)	**Pectoral (Radials)**	n.c.	n.c.	n.c.	n.c.	0.9			0.9		
	**Dorsal**	n.c.	n.c.	n.c.	n.c.	41.9	35.0	16.2	17.1		
	**Anal**	n.c.	n.c.	n.c.	n.c.	1.9				0.7	
**Anomalous shape of fin pterygiophores** (G9*, 8)	**Caudal (Parahypural** 2 **)**	n.c.	n.c.	n.c.	n.c.						0.0
	**Pectoral (Radials)**					13.3		3.8	0.9	5.2	1.6
	**Dorsal**	63.6	15.1	7.3	6.6	52.4	40.0	23.8	22.9	9.0	9.5
	**Anal**	36.4	3.0	4.9	2.5	10.5	10.0	8.6	8.6	7.5	5.6

When the observed value is 0, the cell has been left empty to make easier the reading.

*N* = number of individuals considered, per lot; *n.c.* = data not collected; codes in brackets indicate the anomalies considered (see Table 2).

1 hypurals are reported to ossify endochondrally in zebrafish (*Danio rerio*) [78]–[80], in Senegalese sole (*Solea senegalensis*) [80], in gilthead seabream [81] but other authors found that ossification is perichondral in Nile tilapia and desert pupfish [82];

2 parahypural (a ventral support of caudal fin) is reported [82] as undergoing perichondral ossification, but also that it ossifies endochondrally [71].

**Table 10:** Frequencies (%) of individuals with altered counts, individuals with deformed shape and individuals with both altered counts and deformed shapes of bones undergoing intramembranous (direct) ossification, per lot.

		Group 1		Group 2			Group 3			Group 4	
		INIT06	LVIT01	INIT07	LVIT02	INIT19	LVIT04	INIT18	LVIT05	INGR01	MEGR04
**N**		55	66	123	121	105	40	105	105	134	126
Anomalous number of vertebrae*		16.4		3.2		1.9		4.8			
**Anomalous number and shape of vertebral bodies** (A3/A3*, B3/B3*, C3/C3*, D3/D3*)		12.7		1.6		1.9		1.9			
**Anomalous shape of vertebral body** (S, 1, 2, 3, 3*, 4)		52.7	31.8	17.9	9.9	16.2	10.0	37.1	8.6	17.9	16.7
**Anomalous number of fin rays**	**Caudal**	14.5		34.1	13.2	41.9	10.0	18.1	3.8	29.1	7.9
	**Pectoral**			4.1		5.7	5.0	4.8	1.9	47.0	35.7
	**Dorsal**	3.6		1.6	0.8	7.6		1.9	0.9		
	**Anal**					2.9		0.9			1.6
	**Pelvic**	n.c.	n.c.			0.9				1.5	
**Anomalous number and shape of fin rays**	**Caudal**	5.4		6.5	4.1	1.9	2.5	3.8		1.5	1.6
	**Pectoral**					0.9				13.4	15.1
	**Dorsal**				0.8						
	**Anal**					1.9		0.9			
	**Pelvic**	n.c.	n.c.			0.9					
**Anomalous shape of fin rays**	**Caudal**	20.0		13.8	5.8	4.8	2.5	3.8		9.0	9.5
	**Pectoral**	10.9					2.5		0.9	23.1	28.6
	**Dorsal**	25.4	13.6	19.5	19.0	0.9		13.3	0.9	20.1	12.7
	**Anal**	3.6	4.5	2.4	3.3	15.2	2.5	8.6	0.9	3.7	0.8
	**Pelvic**									5.2	0.8
**Anomalous shape of cranial bone** (16, 17, 17*)		18.2	16.6	21.9	6.6	19.0	5.0	10.5	11.4	3.7	
**Anomalous shape of maxillary** 1 **and dentary** 1 **^,^** 2 (14, 15)		29.1	15.1	33.3	37.7	1.9				11.2	12.7

When the observed value is 0, the cell has been left empty to make reading easier.

*N* = number of individuals considered, per lot; *n.c.* = data not collected; codes in brackets indicate the anomalies considered (see Table 2).

1 [83];

2 [81] reports that dentary ossifies endochondrally but being the only reference affirming this, this bone has been inserted among the intramembranously ossifying elements.

**Table 11:** SWOT analysis of Large Volumes methodology for rearing finfish larvae.

STRENGTHS	WEAKNESSES	OPPORTUNITIES	THREATS
High morphological quality of juveniles	Space availability	Development of local hatchery for niche productions	Competition with intensive hatcheries production
No use of drugs	Skilled operators	Organic hatchery	Lack of rules for juveniles in the established protocols for organic aquaculture
Biofiltering system elimination	Intensive labour for cleaning	Production of “wild like” juveniles better performing for sea-ranching action	Maintenance of wild behaviour in juveniles of top predator species
Expected increasing values for high quality juveniles	Low demand for quality	Higher quality of commercial size fish	Low willingness to pay quality

**Figure 2:**
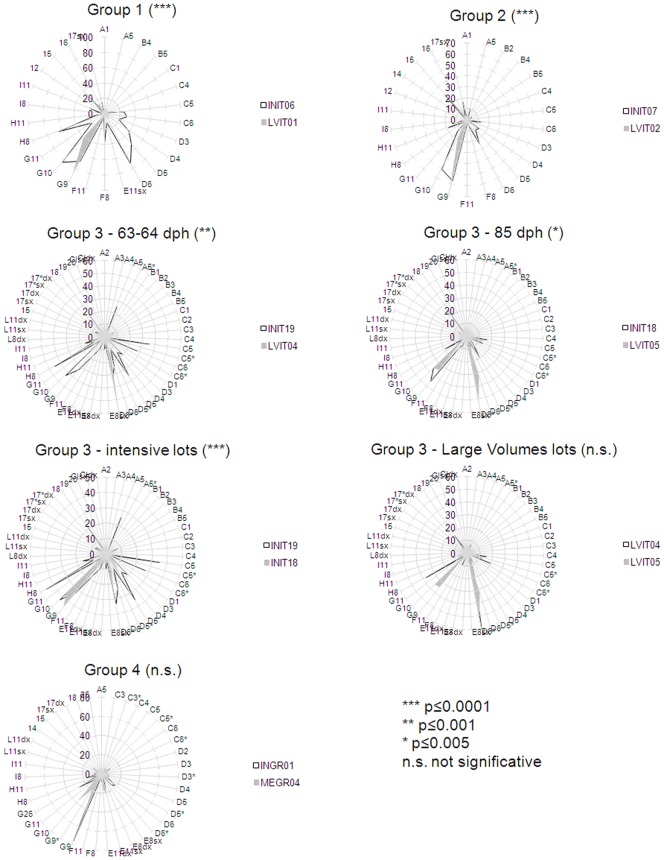
Radar plots showing frequencies of individuals affected by each anomaly, per each group lot. Group 3 is represented with 4 radar plots: two for the comparison between intensive (INIT19, INIT18) and semi-intensive (LVIT04, LVIT05) siblings at 63–64 dph and at 85 dph; the other two compare intensive lots and semi-intensive lots at different ages, respectively. *** = p≤0.0001; ** = p≤0.001; * = p≤0.005; n.s. = not significant.

In order to graphically summarize the main intra-case differences, , the radar plots based on the incidences of individuals found to be affected by each anomaly typology are shown In Fig. 2 in this parameter, and the significance levels (NPMANOVA) of observed differences. All the semi-intensively reared lots were found to be significantly different from the corresponding sister lot reared in intensive conditions. In particular, the comparison among the Group 3 lots showed that the differences between lots reared under the same methodology at a different age (63–64 DAH vs 85 DAH) were highly (p≤0.00000) significant between the two intensive lots (INIT19 vs INIT18), but not between the two Large Volumes lots (LVIT04 vs LVIT05).

The comparison of values obtained in sister lots evidenced that:

- the frequency (%) of individuals with at least one (severe or slight) anomaly was found to be lower in some semi-intensively reared juveniles, e.g., in LVIT04 (Group 3) and MEGR04 (Group 4) lots; in the other groups, either comparable (Group 1) or higher percentages (Group 2 and Group 3/LVIT05) were found;
- the average anomalies load was lower in all the semi-intensive lots compared with sister lots reared in intensive conditions. The only exception is found in Group 4;
- the frequency (%) of individuals with at least one severe anomaly was lower in all the semi-intensively reared lots than in intensively reared sister juveniles;
- the average load of severe anomalies on each individual was lower in all the semi-intensively reared lots but not in LVIT04 (Group 3);
- the severe anomalies/total anomalies ratio observed in each group increased in semi-intensively reared juveniles belonging to Group 1 and in LVIT04 (Group 3), and diminished in all the other groups.

A Correspondence Analysis (CA) was first applied to both BM (981 individuals×41 typologies of anomalies, including ABS) and FM (10 lots×40 typologies of anomalies, without ABS) matrices. As far as the BM is concerned, the resulting ordination of the four sisters groups in the space defined by the first three correspondence axes, CA1, CA2 and CA3, explained 9.9%, 4.7% and 4.2%, respectively, of the overall variance and no interpretable pattern was found in the ordination, which has therefore not been shown.

**Figure 3:**
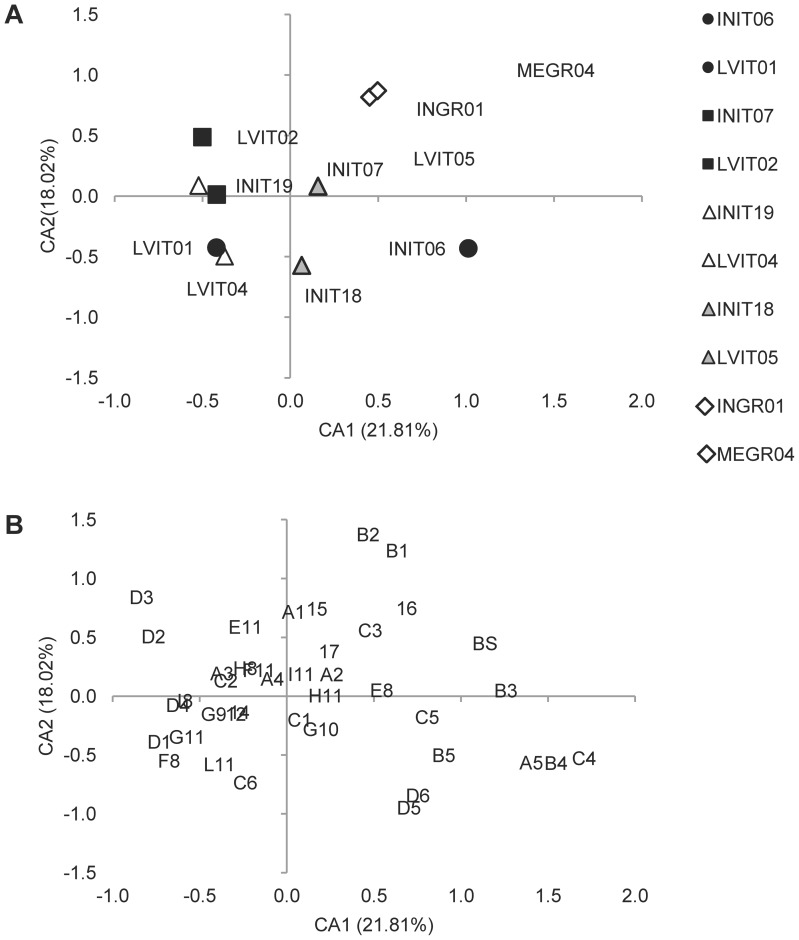
Correspondence Analysis results: ordination model on the first two axes.

**Figure 4:**
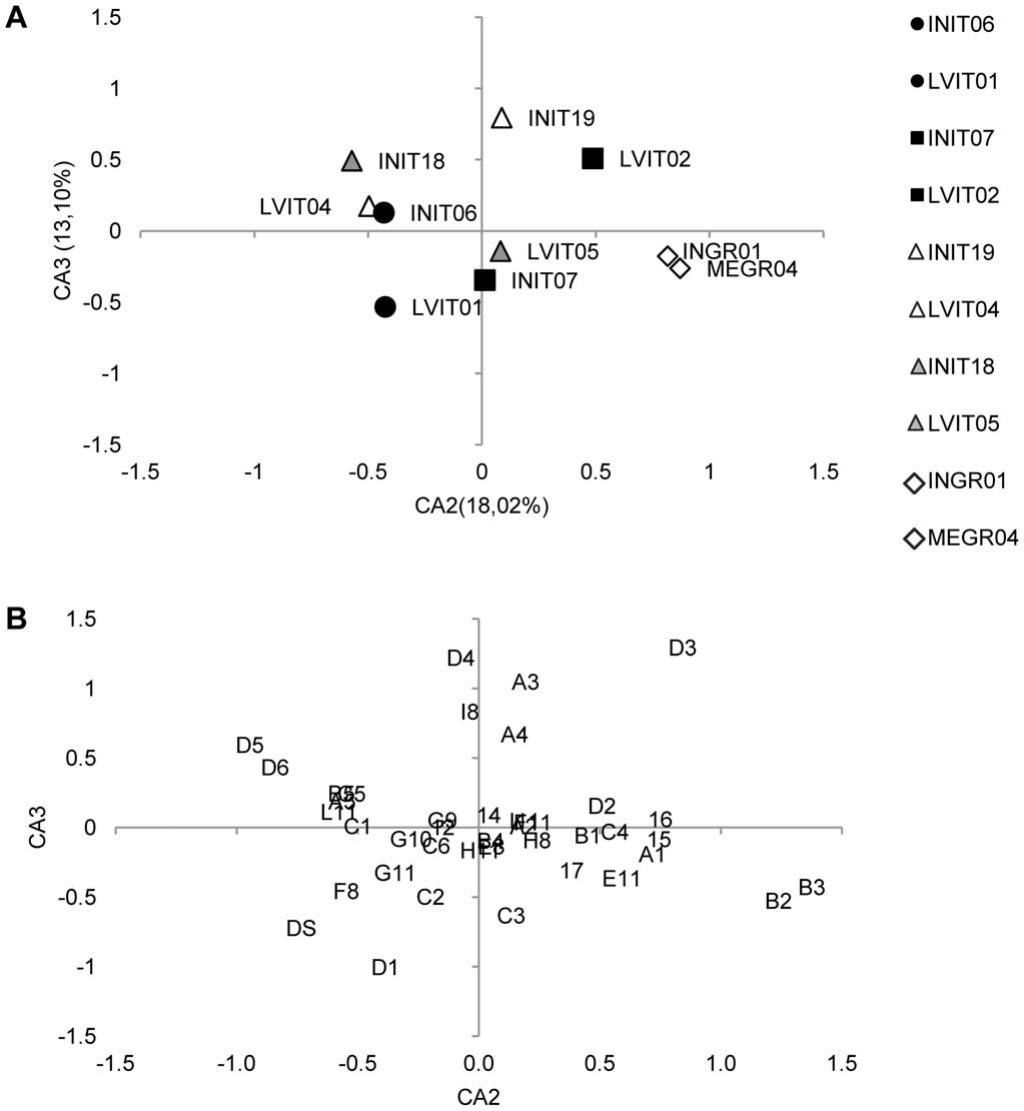
Correspondence Analysis results: ordination model on the 2^nd^ and the 3^rd^ axes.

The CA was then applied to the FM matrix (10 lots×40 typologies of anomalies, without ABS) and the ordination model obtained on the first two correspondence axes is shown in Fig. 3A, whereas the corresponding ordination of descriptors is shown in Fig. 3B. The overall variance explained by the first three axes was 52.9%. The main result is that intensively and semi-intensively reared siblings did not all locate in the same separate space for each rearing methodology, thus evidencing the absence of a commonly-shared pattern of skeletal anomalies in gilthead seabream juveniles reared following a similar methodology. Indeed, only 3 out of 4 Large Volumes reared lots are located on the negative side of CA1 (2^nd^ and 3^rd^ quadrants), whereas the 4^th^ Large Volumes lot and the Mesocosm lot (MEGR04) are located in the 1^st^ quadrant (Fig. 4A). In this quadrant, as many as 10 out of 22 severe anomaly typologies are also found (Fig. 4B).

A second important result is that CA was able to discriminate between siblings only when the Large Volumes methodology was the semi-intensive one. For instance, while all the INIT-lots located in quadrants different from those occupied by the corresponding sister LVIT-lots, and the Group 2 lots plotted in the same quadrant but in different positions in the ordination model inherent in the first two axes (Fig. 3A), and in different quadrants in the ordination model inherent in the 2^nd^ and 3^rd^ axes, as shown in Fig. 4A, then the 4^th^ Group lots (MEGR04 and INGR01) were both superimposed in the same location. This indicates that diversification actually did occur in skeletal anomalies depending on the rearing methodology applied, although a shared common phenotype typical of semi-intensive or intensive conditions was not found. Further, the CA application showed that Large Volumes as a semi-intensive rearing methodology was apparently more effective in modulating the onset of skeletal anomalies, thus producing larger differences with siblings reared in intensive conditions than the other one (Mesocosm).

Group 3 was made up of two sibling lots (INIT19 and LVIT04), sampled at 63–64 and again at 85 dph (INI18 and LVIT05). Unfortunately, in group 4 some technical limitations prevented the same comparison being made between older individuals. The comparison among these lots was used to obtain some indications regarding the fate of anomalies observed one month after the end of the hatchery phase. The ordination model associated with the 2^nd^ and 3^rd^ CA axes (Fig. 4A and B) clearly shows that the distance observed between the two lots at 64 dph is unchanged at 85 dph, although some differences were observed in the skeletal anomalies pattern of older lots, due to the onset, increase or disappearance of some severe anomalies, e.g., opercular plate anomaly (codified as 17) or fused caudal vertebrae bodies (D3) (see Table 8).

## Discussion

This study represents the first attempt to compare the effects of varying rearing methodology on the morphological quality of juveniles of gilthead seabream (*S. aurata*), in many replicae and on a commercial scale: larvae from the same egg batch were split into two lots and reared in intensive (total juveniles observed: 522) or semi-intensive conditions (n = 459), in different farms, periods and countries. Also the relative capacity to improve the morphological quality of two different semi-intensive conditions was tested with respect to corresponding sibling lots reared according to conventional (intensive) methodology. The observation of pattern and frequencies of skeletal anomalies occurring in reared fish at a commercial (and not experimental) level is particularly important, because in this way, the multilevel factors interacting with developing organisms in an unknown way, as happens in farming conditions, and that are not reproducible under experimental conditions, can be included in our approach. Most of the experimental approaches reported in literature for investigating the effect of one factor at a time on the skeletal development of reared fish to our knowledge had actually failed to produce the same anomaly as that observed in rearing conditions.

The results obtained in this study evidenced a significant improvement of the morphological quality (i.e., lowered incidence of severe skeletal anomalies and meristic count variability) of gilthead seabream juveniles reared under semi-intensive conditions, with respect to the sister lots reared with conventional (intensive) methodology (although they failed to identify a clear-cut intensive or semi-intensive typical phenotype based on skeletal anomalies). This datum is particularly interesting because it illustrates how it is possible to ameliorate the morphological quality of reared gilthead seabream juveniles by acting on water volumes, hydrodynamics and administered food preys. The evidence that a wild-like phenotype is approachable but not completely attainable in semi-intensive rearing condition is however inferable from these results.

Literature data on gilthead seabream meristic counts [16],[19]–[20],[63]–[64] reported 24 vertebrae, 13–16 pectoral rays, 10–11 dorsal spines, 11–15 dorsal soft rays, 12–16 anal rays, 9 upper principal caudal rays and 7–8 lower principal caudal rays in wild-caught specimens. This study reported the counts achieved in wild gilthead seabream of all the meristic characters (with the sole exclusion of lateral line scales), thus including also some meristic counts that had never been reported (i.e., predorsal bones) for this species. The 5 wild lots, used here as reference standard, showed a certain range of variation for some characters: 24–25 vertebrae (even if only 1 individual, out of a total of 277, had 25 vertebrae), 3–5 hypurals, 3–4 epurals, 8–10 upper principal caudal rays, 7–8 lower principal caudal rays, 11–14 anal pterygiophores, 13–16 anal rays, 2–3 predorsal bones, 9–11 dorsal spine pterygiophores, 10–12 dorsal spines, 12–16 dorsal soft ray pterygiophores, 13–17 dorsal soft rays, 13–17 pectoral rays, 4 pectoral radials and 6–7 pelvic rays. As far as reared juveniles are concerned, the meristic count variability was higher than that observed in wild lots, thus confirming previous findings for other reared species [4], [65]–[66]; further, the highest variability was found in intensive lots, even if limited to a few individuals or to a few meristic characters. A very interesting datum is that referring to the number of vertebrae in juveniles reared in intensive conditions, where the observed range varies between 23 and 26 vertebrae, while in sister lots reared in semi-intensive conditions (and in wild specimens) the range is 24–25. The number of vertebrae is defined very early during ontogenesis, at the eye-stage embryo (unlike the number of fin rays that are still susceptible to variation after hatching), during the early larval period [67]–[68]. Each Group of lots utilised in this study represents an egg batch that was split into two lots only after hatching, one destined to be reared in intensive conditions, the other in semi-intensive conditions. So far, no differences have been found in the conditions in which the embryonic development occurred, as each egg batch was subject to the same incubation conditions in each Group. Consequently, the differences in vertebral counts observed between intensive and semi-intensive sibling lots of each Group cannot be attributed to different conditions present during egg incubation, but to differences arising later, after hatching. This hypothesis was confirmed by the analysis of frequency of individuals with 23 vertebrae and contemporaneously displaying vertebrae fusions (total and partial) and/or anomalies or axis deviations: 62% of intensively reared juveniles with 23 vertebrae were found to have also vertebral anomalies. The 4 individuals with 26 vertebrae were found only in the INGR01 lot belonging to Group 4: this group, originating from the island of Crete, in the South of Greece, was characterized by the highest frequencies of individuals with 25 (79.1% in INGR01 and 66.7% in MEGR04) instead of 24 vertebrae (the most frequently represented number of vertebrae in wild gilthead seabream) and the only one where as many as 26 vertebrae have been counted. It may thus be postulated that the trend towards a higher number of vertebrae could characterize this South-East Mediterranean group of juveniles, but the fact that no wild gilthead seabream from the Turkish lot (WITU01) showed more or less than 24 vertebrae apparently does not support this hypothesis.

The observation that when meristic count variability increases so does the occurrence of skeletal anomalies confirms what has been previously reported [69] in the same species for a larger number of juveniles, subadults and adults.

In the present study, the incidence of individuals with at least one skeletal (severe or light) anomaly exceeded 80% in all the analyzed lots, but the incidence of individuals with at least one severe anomaly was higher in intensive than in semi-intensive conditions in all groups.

The region found to be most strongly affected in all the lots in the present study was the caudal (vertebrae and fin) one, thus confirming what was previously reported by some authors for this species [16],[70]–[71]. The fact that this region is more susceptible than the other ones to anomalies has not been documented in European seabass (*Dicentrarchus labrax*) or in other reared Teleosts, thus supporting the hypothesis previously advanced [16] that “caudal region sensitivity” could be a species-specific feature in gilthead seabream.

Lordosis and kyphosis were observed in only a few individuals, regardless of the rearing methodology applied, and not in association with the absence of a functional swim bladder (thus confirming [72]–[76]): only 2 juveniles were found with non-inflated swim bladders, but none of them displayed axis deviation. The hydrodynamics inside the tank has been considered to be another important causative factor for haemal lordosis [7], [77]–[79], although in the present study, this anomaly (C2) was found in only 1 individual in the intensive lot INIT19. However, axis deviations generally affected so few and randomly distributed individuals that they could be considered as “background noise” in this study.

In order to understand why and how skeletal anomalies arise, in the present paper the trend of meristic count variation and deformed individual rates was analysed, taking into consideration the ossification typologies of the skeletal elements affected. At least three ossification processes have been described in fish skeletal tissues: *intramembranous ossification*, in which the type of bone (dermal bone) is not preformed into a cartilage, but all these dermal skeleton elements form in the mesenchyme or in the dermis, below a multilayered epithelium or epidermis (majority of exoskeleton); *endochondral ossification*, where a cartilaginous scaffold is replaced by bone (majority of the endoskeleton); *perichondral ossification*, where a cartilaginous precursor is present and usually starts with the transformation of a perichondrium into a periosteum. A few problems were encountered in attempting to correctly assign each bone to the corresponding ossification modality in gilthead seabream owing the presence in literature of many contradictory data: i.e., hypurals are reported to ossify endochondrally in zebrafish (*Danio rerio*) [78]–[80] and, in Senegalese sole (*Solea senegalensis*) [80] and in gilthead seabream [81], whilst other authors [82] described hypural perichondral ossification in Nile tilapia (*Oreochromis niloticus*) and desert pupfish (*Cyprinodon macularis*). Even dentary and opercular series bones are widely reported as intramembranously ossifying in fish (all originating from the neural crest) [83], whilst [81] reported that in gilthead seabream they ossify endochondrally. Finally, endochondral ossification in fish is considered a secondary process of ossification, observable only in fish at more advanced stages or in species with large individuals: in fish larvae essentially only perichondral and no endochondral bone formation occurs ([84]–[85]). In order to obviate any erroneous assignment, in this study only two main ossification modalities have been chosen for consideration, i.e., with (indirect ossification: grouping endochondral and perichondral ossification, Table 9) and without a cartilaginous template (replacement bones, directly ossifying through intramembranous ossification, Table 10), with the bones assigned according to the largest number of available bibliographic reports on fish. The results obtained (see Tabs. 9 and 10) evidenced two different trends: i) in all the semi-intensive lots, the bones having undergone intramembranous ossification showed a constant lower incidence of anomalies; ii) skeletal elements ossifying on a pre-existing cartilaginous template did not always exhibit the same clear pattern, for instance showing a lower incidence of anomalies and lower count variability in all the Large Volumes lots but not in Mesocosm juveniles (MEGR04) (Table 9).

The frequency of individuals affected by severe anomalies was always significantly (NPMANOVA) higher in intensive than in semi-intensive siblings, but the Large Volumes condition showed a some higher, even if not significative, capacity than the Mesocosm one for increasing the gap with the siblings reared in intensive modality, as evidenced by the CA results. This discrepancy between NPMANOVA and CA results could be attributed to the fact that while NPMANOVA analysed differences in quantity (%) of individuals found to be affected by each anomaly, CA analysed the pattern (co-occurrence of some anomalies, i.e.) of skeletal anomalies in the different lots: this means that semi-intensive rearing conditions, whatever they were, induced a significantly lower rate of severely deformed individuals, while only Large Volumes affected also the pattern of skeletal anomalies (different co-occurrence of some anomalies). Further, the results obtained evidenced also that the significant differences between Large Volumes and the corresponding intensive reared sibling persisted even in older individuals (Group 3, 85 dph), but this could not be confirmed in Mesocosm for the lack of sampled older individuals.

The capacity of the Large Volume lot to affect not only the incidence, but also the co-occurrence of severe skeletal anomalies could be ascribed to some nutritional factor: one important difference between Mesocosm and Large Volumes methodologies is the fact that in the latter the input of wild plankton in the tank is routinely practiced, which results in a larger availability for the larvae of different sized preys, with diverse behaviour and nutritional contents. Several vitamins, minerals and dietary lipids are acknowledged to influence the incidence of deformed fishes and it is an accepted fact that natural marine preys for fish larvae, i.e. copepods, have a high content of phospholipids rich in n-3 highly unsaturated fatty acids (HUFA) [86]. As far as gilthead seabream is concerned, [87] found that while an increased dietary HUFA (in particular, docosahexaenoic acid, DHA) level determines an augmentation of anomalies in all skeletal elements with a cartilaginous precursor (vertebral arches), directly mineralizing bones (vertebrae bodies) were found to be unaffected. This could be because increased dietary DHA appreciably increased the risk of peroxidation, whose products (free radicals and oxidised compounds) were found to induce apoptosis, and reactive oxygen species are known to actively destroy cartilage tissue in mammalian bone cells [88]. The negative effects of high dietary DHA in gilthead seabream larvae were significantly reduced by adding Vitamin E to the enriched diet [87]. Vitamin E is an antioxidant that stops the production of reactive oxygen species forming when fat undergoes oxidation. Also dietary Vitamin A differently affects skeletal structure in another advanced Teleost, the Senegalese sole (*Solea senegalensis*), to an extent dependent on the ossification process through which different skeletal structures are derived: those directly originating from the connective tissue with a preliminary cartilage stage were more sensitive to dietary Vitamin A excess than those formed by intramembranous ossification [41]. Therefore, in this scenario, differences in Vitamin or HUFA presence or level in the wide-range preys offered to Large Volumes reared larvae could be responsible for the greater differences observed with corresponding siblings reared in intensive conditions compared with the Mesocosm ones.

The semi-intensive rearing methodologies tested both proved to be capable of significantly ameliorating the morphological quality of juveniles. The Large Volumes methodology also demonstrated the capacity of influencing further growth of juveniles: the comparison between the two samplings performed at two different ages in Group 3 evidenced that Large Volumes morphological quality did not significantly differ in individuals sampled at 64 and 85 dph. It could be debated that 21 days are not sufficient to evidence differences in deformed individual occurrence, but the presence of highly significant differences between the intensive 63 and 85 dph siblings contradicts this. Evidently, the environmental conditions experienced by larvae during Large Volumes rearing are particularly appropriate at least for the differentiation and remodelling of skeletal elements, thus reducing the possibility of developmental anomaly onset or aggravation. It is possible to hypothesize the presence of a higher developmental stability in Large Volumes rearing conditions for gilthead seabream.

As far as density effects on skeletal anomaly onset are concerned, in this study 3 different initial stocking densities were tested: 16 larvae/L in LV lots, 3–4 larvae/L in Mesocosm and 100 larvae/L in all the intensive conditions. Results showed that lowering the stocking densities to less than 16 larvae/L is not in itself a decisive factor for reducing skeletal anomaly incidence, as LV lots were shown to be more or less equally effective in reducing anomaly incidence compared with the mesocosm lot.

In conclusion, the results obtained in this study highlighted that it is possible to ameliorate the morphological quality (i.e., by lowering the severe skeletal anomaly incidence and the meristic count variability of dermal bones) of reared gilthead seabream juveniles by lowering the stocking densities (maximum 16 larvae/L), enlarging the volume of the rearing tanks in the hatchery (minimum 40 m^3^) and feeding larvae with a wide variety of live (wild) preys. Further, the analysis of the morphological quality of juveniles reared under two different semi-intensive conditions, the Mesocosm (*sensu* [46]) and the Large Volumes (*sensu* [47]–[48]), highlighted a greater capacity of Large Volumes to significantly increase the gap with siblings reared in an intensive (conventional) modality.

In Table 11 a SWOT analysis of the Large Volumes rearing methodology is summarized: no use of drugs, biofiltering system elimination (only the use of size filtering and no UVA or ozone treatment), production of high quality juveniles that are particularly good-performing for ongrowning in extensive conditions, and for niche production (local production of autochthonous strains) are the major strengths of this methodology. They also could be successfully applied for the pilot scale rising of larvae of new species for aquaculture, before the development of relative rearing technologies. The Large Volumes approach is a controlled system, developed to recreate natural environmental conditions as closely as possible, with particular regard to prey availability. In this way, tanks and hydrodynamics represent ecological mesocosms [89], in which natural nursery conditions (hydrodynamics, environment diversity and prey availability) are simulated [16]. The simulation of natural conditions is generally achieved by the connection of rearing tanks to external natural water basins (lagoons or ponds), where a natural zooplankton community is present. This causes a natural colonization of the tanks by wild zooplankton (copepod nauplii, juveniles and adults, bivalve trochophores and polychaete larvae), resulting in the constant availability of natural preys, which play an important trophic role, both in terms of energy supply and the learning of feeding behaviour [48],[53]. For this reason, juveniles from Large Volumes rearing maintain a wild behaviour and should be considered as more performing in wild conditions (i.e. restocking or sea-ranching). This issue represents a key opportunity for the production of juveniles destined to become seed in confined coastal lagoon or for sea-restocking actions although it is hard to manage during larval rearing (weakness). The combination with restocking and sea-ranching actions is, together with the use of Large Volumes for organic hatchery or local ‘niche’ hatchery for limited high quality production, the main opportunity that this approach offers. Also the application to innovative species for aquaculture or to species whose larval rearing in intensive conditions is unsatisfactory represents another opportunity. Some experimental Large Volumes tanks (like the Valli Figheri ones, where the juveniles analysed in this study come from) were housed in a plastic greenhouse, where ambient temperature, and not that of tank water, is controlled, with consequent lower heating costs. The main goal of this approach is the production of juveniles of high morphological quality, and immunologically competent toward the most common pathogens thanks to the early and continuous exposition to water containing wild organisms (including the bacterial community).

The major weaknesses are the need for large space availability, the long time consuming learning of know-how, a certain degree of system instability after the 50^th^ dph and a limited production capacity. However, our experience illustrated how in more than 10 years of Large Volumes rearing, an average production of 200,000 European seabass and 120,000 gilthead seabream (80–90 dph) was recorded for each 60 m^3^ tank (Cataudella, pers. comm.). In Large Volumes, water inlet is only size-filtered and no UVA or ozone treatment is applied: this involves that an intensive labour for cleaning is highly required: this may represent a weakness. However, while some early mortality may be expected, all the survivors can be considered as immunologically competent juveniles, and less prone to bacterial infections. A selective mortality based on the most deformed larvae and juveniles cannot be excluded.

As far as economical issues are concerned, both the semi-intensive rearing methodologies tested in this study demonstrated to be capable of ameliorating the morphological quality of gilthead seabream juveniles, with respect to the intensively reared siblings. Higher quality juveniles would mean commercial size fish of some higher quality, if properly ongrown. Thus, an expected increasing value should be recognized to the production of high quality juveniles but at present the willingness to pay higher quality fish seed is still low (also due to the availability of fish seed intensively produced).

## References

[1] Koumoundourous G, Oran G, Divanach P, Stefanakis S, Kentouri M (1997) The opercular complex deformity in intensive gilthead sea bream (*Sparus aurata* L.) larviculture. Moment of apparition and description. Aquaculture 156: 165–177.

[2] Boglione C, Costa C (2011) Skeletal deformities and juvenile quality. In: Pavlidies MA, Mylonas CC, editors. Sparidae. Biology and Aquaculture of Gilthead Seabream and other Species. Wiley-Blackwell Chichester. pp. 233–294.

[3] Paperna I, Colorni A, Gordin H, Kissil GW (1977) Disease of *Sparus aurata* in marine culture at Elat. Aquaculture 10: 195–213.

[4] Matsuoka M (1987) Developmental of skeletal tissues and skeletal muscles in the red sea bream. Bull. Seikai Reg Fish Res Lab 65: 1–114.

[5] Jofre J (1988) Aspectos generales de patologia in fecciosa. In: CAICYT, editors. Patologia en Acuicultura. pp. 1–29.

[6] Balbelona MC, Morinigo MA, Andrades JA, Santamaria JA, Becerra J, et al (1993) Microbiological study of gilthead sea bream (*S. aurata* L.) affected by lordosis (a skeletal deformity). Bull Eur Assoc Fish Pathol 13: 33–36.

[7] Andrades JA, Becerra J, Fernandez-Llebrez P (1996) Skeletal doformities in larval, juvenile and adult stages of cultured gilthead sea bream (*Sparus aurata* L.). Aquaculture 141: 1–11.

[8] Hilomen-Garcia GV (1997) Morphological abnormalities in hatchery-bred milkfish (*Chanos chanos* Forsskal) fry and juveniles. Aquaculture 152: 155–166.

[9] Haga Y, Suzuki T, Takeuchi T (2002) Retinoic acid isomers produce malformations in postembryonic development of Japanese flounder, *Paralichthys olivaceus* . Zool Sci 19: 1105–1112. 12426472 10.2108/zsj.19.1105

[10] Imsland AK, Foss A, Koedjik R, Folkvord A, Stefansson SO, et al (2006) Short- and long-term differences in growth, feed conversion efficiency and deformities in juvenile Atlantic cod (*Gadus morhua*) start fed on rotifers or zooplankton. Aquac Res 37: 1015–1027.

[11] Le Vay L, Carvalho GR, Quinitio ET, Lebata JH, Ut VN, et al (2007) Quality of hatchery-reared juveniles for marine fisheries stock enhancement. Aquaculture 268: 169–180.

[12] Puvanendran V, Calder-Crewe C, Brown JA (2009) Vertebral deformity in cultured Atlantic cod larvae: ontogeny and effects on mortality. Aquac Res 40: 1653–1660.

[13] Boglione C, Gavaia P, Koumoundouros G, Gisbert E, Moren M, et al (2012a) A review on skeletal anomalies in reared European fish larvae and juveniles. Part 1: normal and anomalous skeletogenic processes. Reviews in Aquaculture in press.

[14] Boglione C, Gisbert E, Gavaia P, Witten PE, Moren M, et al (2012b) A review on skeletal anomalies in reared European fish larvae and juveniles. Part 2: main typologies, occurrences and causative factors. Reviews in Aquaculture in press.

[15] Favaloro E, Mazzola A (2000) Meristic character analysis and skeletal anomalies during growth in reared sharpsnout seabream. Aquac Int 8: 417–430.

[16] Boglione C, Gagliardi F, Scardi M, Cataudella S (2001) Skeletal descriptors and quality assessment in larvae and post-larvae of wild-caught and hatchery-reared gilthead sea bream (*Sparus aurata* L. 1758). Aquaculture 192: 1–22.

[17] Boglione C, Costa C, Di Dato P, Ferzini G, Scardi M, et al (2003) Skeletal quality assessment of reared and wild sharpsnout sea bream and pandora juveniles. Aquaculture 227: 373–394.

[18] Boglione C, Marino G, Giganti M, Longobardi A, De Marzi P, et al (2009) Skeletal anomalies in dusky grouper *Epinephelus marginatus* (Lowe 1834) juveniles reared with different methodologies and larval densities. Aquaculture 291: 48–60.

[19] Faustino M, Power DM (1998) Development of osteological structures in the sea bream: vertebral column and caudal fin complex. Journal of Fish Biology 52: 11–22.

[20] Faustino M, Power DM (1999) Development of the pectoral, pelvic, dorsal and anal fins in cultured sea bream. Journal of Fish Biology 54: 1094–1110.

[21] Cahu C, Zambonino Infante J, Takeuchi T (2003) Nutritional components affecting skeletal development in fish larvae. Aquaculture 227: 245–258.

[22] Matsuoka M (2003) Comparison of meristic variations and bone abnormalities between wild and laboratory-reared red sea bream. Jarq 37: 21–30.

[23] Lall SP, Lewis-McCrea LM (2007) Role of nutrient in skeletal metabolism and pathology in fish – An overview. Aquaculture 267: 3–19.

[24] Castro J, Pino-Querido A, Hermida M, Chavarrias D, Romero R, et al (2008) Heritability of skeleton abnormalities (lordosis, lack of operculum) in gilthead seabream (*Sparus aurata*) supported by microsatellite family data. Aquaculture 279: 18–22.

[25] Costa C, Vandeputte M, Antonucci F, Boglione C, Menesatti P, et al (2010) Genetic and environmental influences on shape variation in the European sea bass (*Dicentrarchus labrax*). Biological. Journal of the Linnean Society 101: 427–436.

[26] Allaby M (2004) Oxford Dictionary of Ecology. Oxford University Press Inc., New York p. 201.

[27] Almeida D, Almodóvar A, Nicola GG, Elvira B (2008) Fluctuating asymmetry, abnormalities and parasitism as indicators of environmental stress in cultured stocks of goldfish and carp. Aquaculture 279: 120–125.

[28] Favaloro E, Mazzola A (2003) Meristic variation and skeletal anomalies of wild and reared sharpsnout seabream juveniles (*Diplodus puntazzo*, Cetti 1777) off coastal Sicily, Mediterranean Sea. Fish Physiology and Biochemistry 32: 159–166.

[29] Favaloro E, Mazzola A (2006) Meristic character counts and incidence of skeletal anomalies in the wild *Diplodus puntazzo* (Cetti, 1777) of an area of the south-eastern Mediterranean Sea. Fish Physiology and Biochemistry 32: 159–166.

[30] Sfakianakis DG, Doxa CK, Kouttouki S, Koumoundouros G, Maingot E (2005) Osteological development of the vertebral column and of the fins in *Diplodus puntazzo* (Cetti, 1777). Aquaculture 250: 36–46.

[31] Kouttouki S, Georgakopoilou E, Kaspiris P, Divanach P, Koumoundouros G (2006) Shape ontogeny and variation in the sharpsnout seabream, *Diplodus puntazzo* (Cetti, 1777). Aquaculture Research 37: 655–663.

[32] Verhaegen Y, Adriaens D, De Wolf T, Dhert P, Sorgeloos P (2007) Deformities in larval gilthead sea bream (*Sparus aurata*): A qualitative and quantitative analysis using geometric morphometrics. Aquaculture 268: 158–168.

[33] Koumoundourous G (2008) First record of saddleback syndrome in wild parrotfish *Sparisoma cretense* (L. 1758) (Perciformes, Scaridae). Journal of Fish Biology 72: 737–741.

[34] Boglione C, Costa C, Giganti M, Di Dato P, Scardi M, et al (2006) Biological monitoring of wild thicklip grey mullet (*Chelon labrosus*), golden grey mullet (*Liza aurata*), thinlip mullet (*Liza ramada*) and flathead mullet (*Mugil cephalus*) (Pisces: Mugilidae) from different Adriatic sites: Meristic counts and skeletal anomalies. Ecol Indic 6: 712–732.

[35] Afonso JM, Montero D, Robaina L, Astorga N, Izquierdo MS, et al (2000) Association of lordosis – scoliosis – kyphosis deformity in gilthead seabream (*Sparus aurata*) with family structure. Fish Physiol Biochem 22: 159–163.

[36] Koumoundouros G, Divanach P, Kentouri M (2001) The effects of rearing conditions on development of saddleback syndrome and caudal fin deformities in *Dentex dentex* . Aquaculture 200: 285–304.

[37] Koumoundouros G, Maingot E, Divanach P, Kentouri M (2002) Kyphosis in reared sea bass (*Dicentrarchus labrax* L.): ontogeny and effects on mortality. Aquaculture 209: 49–58.

[38] Lewis LM, Lall SP, Witten PE (2004) Morphological descriptions of the early stages of spine and vertebral development in hatchery-reared larval and juvenile Atlantic halibut (*Hippoglossus hippoglossus*). Aquaculture 241: 47–59.

[39] Hough C (2009) Manual of control of malformations in fish aquaculture. Science and Practice. In: Baeverfjord G, Helland S, Hough C, editors. Federation of European Aquaculture Producers, RapidPRess (Luxembourg) pp 150. Available: http://www.feap.info/default.asp?SHORTCUT=633. Accessed 2013 Jan 8.

[40] Kause A, Ritola O, Paananen T (2007) Changes in the expression of genetic characteristics across cohorts in skeletal deformations of farmed salmonids. Genetics Selection Evolution 39: 529–543. 10.1186/1297-9686-39-5-529 PMC2682804 17897595

[41] Fernandez I, Gisbert E (2010) Senegalese sole bone tissue originated from chondral ossification is more sensitive than dermal bone to high vitamin A content in enriched *Artemia* . J Appl Ichthyol 26: 344–349.

[42] Koumoundouros G, Gagliardi F, Divanach P, Boglione C, Cataudella S, et al (1997) Normal and abnormal osteological development of caudal fin in *Sparus aurata* L. fry. Aquaculture 149: 215–226.

[43] Hall BK (2005) Bones and Cartilage: Developmental and Evolutionary Skeletal Biology. Elsevier Academis Press, London, UK.

[44] Kentouri M, Divanach P, Sterioti A, Papapetrou M, Maingot E, et al. (1995) A comparative study of cost effectiveness with growth performances of *sea bream Sparus aurata* larvae reared under two different climatic conditions in France and Greece using three types of Mesocosms. Report of the AQ. 2. 429 FAR project.

[45] Dhert P, Divanach P, Kentouri M, Sorgeloos P (1998) Rearing techniques for difficult marine fish larvae. World Aquaculture, March 48–55.

[46] Divanach P, Kentouri M (2000) Hatchery techniques for specific diversification in Mediterranean finfish larviculture. Cahiers options méditerranéennes 47: 75–87.

[47] Cataudella S, Russo T, Cataldi E, Boglione C, Saroglia M (2003) Does the model of semi-intensive larval rearing of Mediterranean finfish contribute to the knowledge on animal welfare in aquaculture? In: Geatti F, Berardo P, Zanchetta S, editors. Book of Abstract, International Aquaculture Conference “Fish farming in Mediterranean Europe: Quality for Developing Markets” Verona, Italy, October 15–16 2003.

[48] Cataudella S, Russo T, Lubrano P, De Marzi P, Spanò A, et al.. (2002) An ecological approach to produce “wild like” juveniles of sea bass and sea bream: trophic ecology in semi-intensive hatchery conditions, in: Seafarming today and tomorrow. Extended abstracts and short communications. Presented at the Aquaculture Europe 2002, Trieste, Italy, October 16–19. pp. 177–178.

[49] Marino G, Boglione C, Cataudella S, Saroglia M, Ingle E (1989) Growth, swim bladder development and body abnormalities of sea bass (*Dicentrarchus labrax* L.) larvae reared with the greenwater technology in large culture volumes, in: European Acquaculture Society Symposium, Bordeaux, October, 2-3-4, Sp Pub10. pp. 313–314.

[50] Spedicato MT, Boglione C (2000) Main constraints in the artificial propagation of *Epinephelus marginatus* (Lowe, 1834): three year experimental trials on induced spawning and larval rearing. Cah Options Méditerr 47: 227–234.

[51] Malavasi S, Georgalas V, Lugli M, Torricelli P, Mainardi D (2004) Differences in the pattern of antipredator behaviour between hatchery-reared and wild sea-bass (*Dicentrarchus labrax*) juveniles. Journal of Fish Biology 65: 143–155.

[52] Georgalas V, Malavasi S, Franzoi P, Torricelli P (2007) Swimming activity and feeding behaviour of larval European sea bass (*Dicentrarchus labrax* L.): effects of ontogeny and increasing food density. Aquaculture 264: 418–427.

[53] Russo T, Costa C, Cataudella S (2007) Correspondence between shape and feeding habit changes throughout ontogeny of gilthead sea bream *Sparus aurata* L., 1758. Journal of Fish Biology 71: 629–656.

[54] Russo T, Boglione C, De Marzi P, Cataudella S (2008) Feeding preferences of the dusky grouper larvae reared in semi-intensive conditions: a contribution addressing the domestication of this species. Aquaculture 289: 289–296.

[55] Russo T, Scardi M, Boglione C, Cataudella S (2010b) Rearing methodologies and morphological quality in aquaculture: an application of the Self – Organizing Map to the study of skeletal anomalies in dusky grouper (*Epinephelus marginatus* Lowe, 1834) juveniles reared under different methodologies. Aquaculture 315: 69–77.

[56] Papandroulakis N, Divanach P, Kentouri M (2002) Enhanced biological performance of intensive sea bream (*Sparus aurata*) larviculture in the presence of phytoplankton with long photophase. Aquaculture 204: 45–63.

[57] Dingerkus G, Uhler LD (1977) Enzyme clearing of Alcian blue stained whole small vertebrates for demonstration of cartilage. Stain Technol 52: 229–232. 71769 10.3109/10520297709116780

[58] Harder W (1975) Anatomy of Fishes. Part I. In: Schweizebart'sche Verlagsbuchhandlung E, editors, Stuttgart. 612 pp.

[59] Schultze H P, Arratia G (1989) The composition of the caudal skeleton of Teleosts (*Actinopterygii: Osteichthyes*). Zool J Linnean Soc 97: 189–231.

[60] Benzecrì JP (1973) L'Analyse des Données. L'Analyse des Correspondances, vol. 2. Dunod, Paris, France. p. 628.

[61] Sokal RR, Michener CD (1958) A statistical method for evaluating systematic relationships. University of Kansas Scientific Bulletin 38: 1409–1438.

[62] Hammer Ø, Harper DAT, Ryan PD (2001) PAST: Paleontological Statistics Software Package for Education and Data Analysis. Palaeontologia Electronica 4: 9.

[63] Bianchi G (1984) Study on the morphology of five Mediterranean and Atlantic Sparid fishes with a reinstatement of the genus *Pagrus*, Cuvier, 1817. Cybium 8: 31–56.

[64] Fischer W, Schneider M, Bauchot ML (Rédacteurs) (1987) Fiches FAO d'identification des espéces pour le besoins de la peche. (Révision 1). Méditerranée et Mer Noire. Zone de peche 37, vol. 2 (Vertébrés). FAO, Rome, Vol. 2. pp. 761–1530.

[65] Boglione C, Bertolini B, Cataudella S, Rossi A, Ferreri F, et al.., (1993) Larval and postlarval monitoring in sea bass: morphological approach to evaluate finfish seed quality. In: Barnabe G, Kestemont P, editors. Production Environment Quality Bordeaux Aquaculture '92, Bordeaux, France, March 25–27, 1992. European Aquaculture Society. Sp. Pub. 18. pp. 189–204.

[66] Marino G, Boglione C, Bertolini B, Rossi A, Ferreri F, et al (1993) Observations on the development and anomalies in the appendicular skeleton of sea bass, *D. labrax* L. 1758, larvae and juveniles. Aquacult Fish Manage 24: 445–456.

[67] Tåning AV (1952) Experimental study of meristic characters in fishes. Biol Rev 27: 169–197.

[68] Fowler LA (1970) Control of vertebral number in teleosts an embryological problem. Quart Rev Biol 45: 148–167.

[69] Russo T, Prestinicola L, Scardi M, Palamara E, Cataudella S, et al (2010) Progress in modeling quality in aquaculture: an application of the Self-Organizing Map to the study of skeletal anomalies and meristic counts in gilthead seabream (*Sparus aurata*, L. 1758). J Appl Ichthyol 26: 360–365.

[70] Koumoundourous G, Gagliardi F, Divanach P, Stefanakis S, Kentouri M (1995) Osteological study on the origin and development of the abnormal caudal fin in gilthead sea bream (*S. aurata*)fry. Quality in Aquaculture. Eur Aquacult Soc, Spec Publ vol. 23 European Aquaculture Society, Gent, Belgium. pp. 16–18, 413.

[71] Fernandez I, Hontoria F, Ortiz-Delgado JB, Kotzamanis Y, Estévez A (2008) Larval performance and skeletal deformities in farmed gilthead sea bream (*Sparus aurata*) fed with graded levels of Vitamin A enriched rotifers (*Brachionus plicatilis*). Aquaculture 283: 102–115.

[72] Paperna I (1978) Swimbladder and skeletal deformations in hatchery bred *Sparus aurata* . Journal of Fish Biology 12: 109–114.

[73] Tesseyre C (1979) Étude des conditions d'élevage intensif du loup (*Dicentrarchus labrax* L.).Thèse de 3^e^ cycle, Université de Montpellier. 115 pp.

[74] Kitajima C, Tsukashima Y, Fujita S, Watanabe T, Yone Y (1981) Relationship between uninflated swimbladder and lordotic deformity in hatchery-reared red sea bream *Pagrus major* . Bull Jpn Sot Sci Fish 47: 1289–l294.

[75] Chatain B (1994) Abnormal swimbladder development and lordosis in sea bass (*Dicentrarchus labrax*) and sea bream (*Sparus aurata*). Aquaculture 119: 371–379.

[76] Boglione C, Cataudella S, Ferreri F, Finoia MG, Fusari A, et al (1995) Skeletal anomalies in *Dicentrarchus labrax* juveniles selected for functional swimbladder. Ices Mar Sci Symp 201: 163–169.

[77] Divanach P, Papaundroulakis N, Anastasiadis P, Koumoundouros G, Kentouri M (1997) Effect of water currents on the development skeletal deformities in sea bass (*Dicentrarchus labrax*) with functional swimbladder during post larval and nursery phase. Aquaculture 156: 145–155.

[78] Bird NC, Mabee PM (2003) Developmental morphology of the axial skeleton of the zebrafish, *Danio rerio* (Ostariophysi: Cyprinidae). Developmental Dynamics 228: 337–357. 14579374 10.1002/dvdy.10387

[79] Bird NC, Hernandez LP (2009) Building an evolutionary innovation: differential growth in the modified vertebral elements of the zebrafish Weberian apparatus. Zoology 112: 97–112. 19027276 10.1016/j.zool.2008.05.003

[80] Gavaia PJ, Simes DC, Ortiz-Delgado JB, Viegas CSB, Pinto JP, et al (2006) Osteocalcin and matrix Gla protein in zebrafish (*Danio rerio*) and Senegal sole (*Solea senegalensis*): comparative gene and protein expression during larval development through adulthood. Gene Expression Patterns 6: 637–652. 16458082 10.1016/j.modgep.2005.11.010

[81] Estêvão DM, Silva N, Redruello B, Costa R, Gregório S, et al (2011) Cellular morphology and markers of cartilage and bone in the marine teleost *Sparus auratus* . Cell and Tissue Research 343: 619–635. 21234603 10.1007/s00441-010-1109-y

[82] Witten PE, Huysseune A (2007) Mechanisms of Chondrogenesis and Osteogenesis in Fins. In: Hall BK (ed.) Fins into Limbs: Evolution, Development, and Transformation, pp. 79–92. The University of Chicago Press, Chicago.

[83] Sire J-Y, Huysseune A (2003) Formation of dermal skeletal and dentary tissues in fish: a comparative and evolutionary approach. Biological Reviews 78: 219–249. 12803422 10.1017/s1464793102006073

[84] Witten PE, Hansen A, Hall BK (2001) Features of Mono- and Multinucleated Bone Resorbing Cells of the Zebrafish *Danio rerio* and Their Contribution to Skeletal Development, Remodeling and Growth. Journal of Morphology 250: 197–207. 11746460 10.1002/jmor.1065

[85] Witten PE, Huysseune A (2010) The unobtrusive majority: mononucleated bone resorbing cells in teleost fish and mammals. Journal of Applied Ichthyology 26: 225–229.

[86] Shields RJ, Bell JG, Luizi FS, Gara B, Bromage NR, et al (1999) Natural copepods are superior to enriched *Artemia* Nauplii as feed for Halibut larvae (*Hippoglossus hippoglossus*) in terms of survival, pigmentation and retinal morphology: Relation to Dietary Essential Fatty Acids. J Nutr vol. 129 no. 6: 1186–1194. 10356085 10.1093/jn/129.6.1186

[87] Izquierdo MS, Scolamacchia M, Betancor M, Roo J, Caballero MJ, et al (2012) Increased dietary docosahexaenoic acid promotes early mineralization in *Sparus aurata* (Linnaeus, 1758) preventing lordosis and kyfosis occurrence, whereas an excess level increases chondroid bones anomalies. British Journal of Nutrition In press.

[88] Beer AM, Wegener T (2011) Vitamin E for gonarthrosis and coxarthrosis: results of a postmarketing surveillance study. Fortschr Med 153: 14–20. 21591326

[89] Beyers RJ, Odum HT (1993) Ecological Microcosms. New York, Springer.

